# Chemical Emissions From Heated Vitamin E Acetate—Insights to Respiratory Risks From Electronic Cigarette Liquid Oil Diluents Used in the Aerosolization of Δ^9^-THC-Containing Products

**DOI:** 10.3389/fpubh.2021.765168

**Published:** 2022-01-21

**Authors:** Ryan F. LeBouf, Anand Ranpara, Jason Ham, Michael Aldridge, Elizabeth Fernandez, Kenneth Williams, Dru A. Burns, Aleksandr B. Stefaniak

**Affiliations:** ^1^Respiratory Health Division, National Institute for Occupational Safety and Health, Morgantown, WV, United States; ^2^Health Effects Laboratory Division, National Institute for Occupational Safety and Health, Morgantown, WV, United States

**Keywords:** e-cigarettes, e-liquids, vitamin E acetate, EVALI, chemical emissions

## Abstract

As of February 18, 2020, the e-cigarette, or vaping, product use associated lung injury (EVALI) outbreak caused the hospitalization of a total of 2,807 patients and claimed 68 lives in the United States. Though investigations have reported a strong association with vitamin E acetate (VEA), evidence from reported EVALI cases is not sufficient to rule out the contribution of other chemicals of concern, including chemicals in either THC or non-THC products. This study characterized chemicals evolved when diluent oils were heated to temperatures that mimic e-cigarette, or vaping, products (EVPs) to investigate production of potentially toxic chemicals that might have caused lung injury. VEA, vitamin E, coconut, and medium chain triglyceride (MCT) oil were each diluted with ethanol and then tested for constituents and impurities using a gas chromatograph mass spectrometer (GC/MS). Undiluted oils were heated at 25°C (control), 150°C, and 250°C in an inert chamber to mimic a range of temperatures indicative of aerosolization from EVPs. Volatilized chemicals were collected using thermal desorption tubes, analyzed using a GC/MS, and identified. Presence of identified chemicals was confirmed using retention time and ion spectra matching with analytic standards. Direct analysis of oils, as received, revealed that VEA and vitamin E were the main constituents of their oils, and coconut and MCT oils were nearly identical having two main constituents: glycerol tricaprylate and 2-(decanoyloxy) propane-1,3-diyl dioctanoate. More chemicals were measured and with greater intensities when diluent oils were heated at 250°C compared to 150°C and 25°C. Vitamin E and coconut/MCT oils produced different chemical emissions. The presence of some identified chemicals is of potential health consequence because many are known respiratory irritants and acute respiratory toxins. Exposure to a mixture of hazardous chemicals may be relevant to the development or exacerbation of EVALI, especially when in concert with physical damage caused by lung deposition of aerosols produced by aerosolizing diluent oils.

## Introduction

The e-cigarette, or vaping, product use associated lung injury (EVALI) outbreak was a public health crisis that caused the hospitalization of 2,807 people and was responsible for 68 deaths in the United States as of February 18, 2020 (1). Inhalation of vitamin E acetate (VEA) is strongly associated with the EVALI cases described during the outbreak (2) and EVALI-like syndromes have been produced in mouse models by VEA (3). Evidence is not sufficient to rule out the contribution of other chemicals of concern, including chemicals in either THC or non-THC products, in some of the reported EVALI cases and mechanisms of lung injury in EVALI are incompletely understood (4). Blount et al. (2) hypothesize one possible mechanism of injury could be that the aliphatic tail of VEA could penetrate a layer of lung surfactant to align the molecule in parallel with phospholipids, thereby interfering with surfactant function. However, all users of VEA-containing e-liquids presumably did not develop EVALI, which may indicate unrecognized risk factors, or a difference in individual susceptibilities to the stressors. Public health messaging, the removal of VEA from electronic cigarette liquid (e-liquid) formulations, and law enforcement practices might have led to the decline in emergency department visits related to e-cigarette, or vaping, products (EVPs) (1). As of January 14, 2020, 82% (1,658/2,022) of hospitalized EVALI patients reported using products containing Δ^9^-tetrahydrocannabinol (THC), 33% reported exclusive use of THC-containing products, 57% reported using any nicotine-containing product, and 14% reported using only nicotine-containing products (1). According to the (5), the cannabis plant is referred to as “marijuana” when the plant contains more than 0.3% by dry weight of Δ^9^-THC, the main psychoactive cannabinoid, and referred to as “hemp” when the plant contains <0.3% of Δ^9^-THC (2018). Marijuana is the most popular illicit/recreational drug in the United States with ~123 million people (age 12 + years) who have tried it at least once in their lifetime and ~41 million people who have used it in the past year (6). In 2014, among adults who reported using marijuana, 9.9% reported using “vaporizers” (devices used for drug delivery via inhalation) or other electronic devices. Of current adult EVP users, 17% reported using marijuana and 70% reported using nicotine (7). Among college students, 75% of those who reported using a substance other than nicotine in EVPs responded that they used marijuana or Δ^9^-THC-containing products (8).

EVPs for nicotine delivery heat an e-liquid composed of propylene glycol and vegetable glycerin. Cannabis extracts are hydrophobic, semi-solid materials. During the time of the EVALI outbreak, illicit Δ^9^-THC products were shown to commonly be diluted with terpenes, VEA, medium chain triglycerides, and polyethylene glycol (9, 10). VEA is strongly associated with EVALI and has been identified in bronchoalveolar lavage specimens from 48 of 51 EVALI cases in 16 states, giving direct evidence at the site of injury (2). Bhat et al. provided additional evidence of lung injury consistent with EVALI by exposing mice to VEA aerosols but recognized the need to characterize chemical emissions from heated VEA (3).

THC oil is aerosolized in EVPs that contain a reservoir with a wick, a heating element, and a battery. For propylene glycol and vegetable glycerin based e-liquids commonly used with nicotine, top-coil or clearomizer-coil temperatures range from 322 to 1,008°C for dry conditions, 145–334°C for wet-through-wick conditions, and 110–185°C for full-wet conditions (11). Lynch et al. observed that temperatures ranged from 375 to 569°C when THC/VEA were aerosolized with ceramic coil cartridges (12). The inhaled aerosol contains liquid droplets that will deposit throughout the respiratory tract based, in part, on their aerodynamic diameter, and gas-phase substances, including volatile organic compounds (VOCs). Hence, it is likely that both the physical (particle size) and chemical constituents of inhaled aerosol have deleterious individual and combined respiratory and systemic effects. For example, VEA, when aerosolized using third-generation EVPs, produces ethenone (C_2_H_2_O) gas, a type of ketene gas and respiratory irritant, which is hypothesized to be a contributing factor to EVALI (13). Attfield et al. also hypothesized that thermal degradation of VEA may be important in EVALI and suggested that acetate moieties are precursors for high temperature formation of ethenone gas. The researchers also note that the reaction could be amplified at high temperatures in the presence of catalytic metals, including chromium and nickel, and/or ceramic surfaces present in heating coils of EVPs. According to Wu & O'Shea (13), additional thermal transformation products produced from aerosolized VEA included benzene, butadiene, and formaldehyde. All of these chemicals are respiratory irritants and potential occupational carcinogens (14). Chemical emissions from heating oil diluents during aerosolization are composed of the unadulterated chemical, thermal breakdown and rearrangement products from the original chemical, and new products formed in the presence of oxygen. In this study, we elucidated several chemical emissions of VEA and other oil diluents as well as assigned relative hazard designations to identified chemicals. This information provides novel insights for investigators to study chemical toxicity in concert with aerosol deposition modeling to better understand the mechanism(s) of lung injury observed among EVALI patients.

## Methods

### Materials

VEA (α-tocopherol acetate), ≥99%, CAS# 7695-91-2, was purchased from Sigma-Aldrich (St. Louis, MO). Vitamin E oil (42900IU, 100% pure & natural, Chandler, AZ), medium chain triglycerides (MCT, 100% organic unflavored, Garden of Life LLC, FL), and coconut oil (Organic unflavored, Carrington Farms, Closter, NJ) were purchased from Amazon.com, Inc. (Seattle, WA).

Chemical standards and reagents were purchased from various suppliers:

Sigma-Aldrich (St. Louis, MO): *O*-2,3,4,5,6-pentafluorobenzyl hydroxylamine hydrochloride (PFBHA), ≥99%, CAS# 57981-02-9; N,N-dimethylbenzamide, CAS# 611-74-5; glyceryl trioctanoate, CAS# 538-23-8; hexamethylcyclotrisiloxane, CAS# 541-05-9; nonanal, CAS# 124-19-6; acetic anhydride, CAS# 108-24-7; isoamyl ether, CAS# 544-01-4; 2,6,10-trimethyldodecane, CAS# 3891-98-3; methylpropenal (methacrolein), CAS# 78-85-3; isovaleraldehyde, CAS# 590-86-3; 1-ethyl-2-pyrrolidinone, CAS# 2687-91-4; undecanoic acid, CAS# 112-37-8; 2-butyl-1-octanol, CAS# 3913-02-8, diacetyl, CAS# 431-03-8, methylglyoxal, CAS# 78-98-8, 2-butanone, CAS# 78-93-3.

Accela ChemBio (San Diego, CA): hexahydrofarnesyl acetone, CAS# 502-69-2; 2-hexyl-1-octene, CAS# 19780-80-4; and 2-ethylcrotonaldehyde, CAS# 19780-25-7.

Alfa-Chemistry (Stony Brook, NY): 2-methylpentyl formate, CAS# 381670-34-4.

Cayman Chemical (Ann Arbor, MI): hexadecanal, CAS# 629-80-1.

Crescent Chemical (Islandia, NY): pristane, CAS# 1921-70-6.

Fisher Scientific (Pittsburgh, PA): acetic acid, CAS# 64-19-7; 1,2-epoxyhexane (Acros Organics), CAS# 1436-34-6; 2-methylheptane(Acros Organics), CAS# 592-27-8; 2-nonanone (Acros Organics), CAS# 821-55-6.

Oakwood Chemical (Estill, SC): 1,2-epoxyoctadecane, CAS# 7390-81-0; 2-methoxyacetic acid, CAS# 625-45-6.

Tokyo Chemical Industry Co. (Montgomeryville, PA): 1-eicosene, CAS# 3452-07-1.

### Bulk Oil Analysis

We performed analysis of oils to evaluate constituents and impurities by diluting with ethanol to 10^−3^, 10^−4^, and 10^−5^. We injected the diluted oil and analyzed using a 7890B/5977B gas chromatograph/mass spectrometer (GC/MS, Agilent Technologies, Inc., Santa Clara, CA). The column was a Restek (Bellefonte, PA) Rxi®-1ms (0.32 mm I.D., 60 m long, 1.0 μm film thickness) with ultra-high purity helium flowing at 2 mL min^−1^. The oven program was 58.25 min long and started at 30°C for 5 min, 5°C min^−1^ to 170°C, 20°C min^−1^ to 250°C, hold for 10 min, 40°C min^−1^ to 300°C, hold for 10 min. Electron impact (EI) ionization spectra were collected from *m/*z 30–500 with an MS source temperature of 300°C and quadrupole temperature of 150°C.

### Experimental Setup and Design

**Figure 1:**
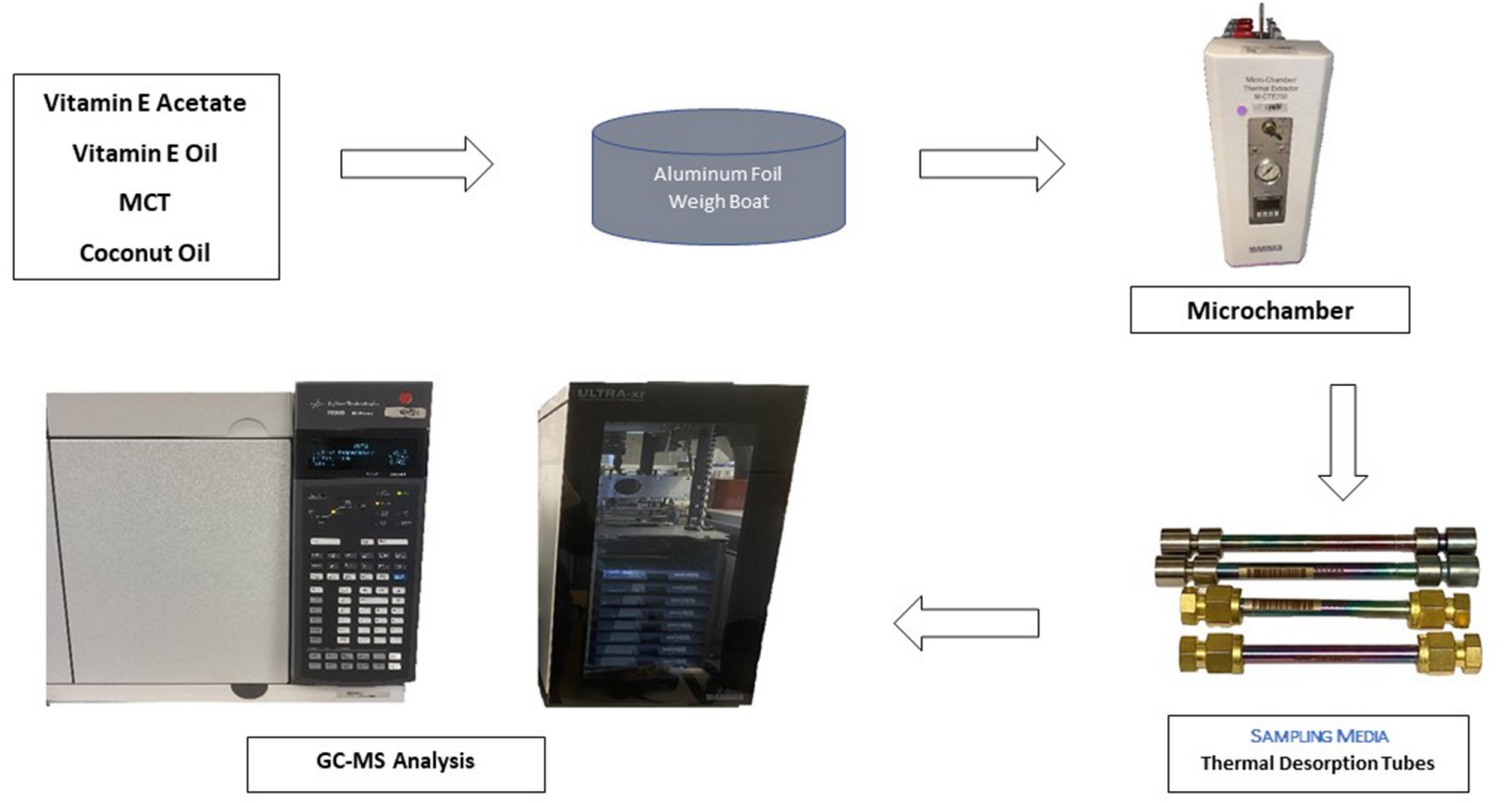
Flow diagram of emission testing of oil diluents.

Aluminum weigh boats (Fischer Scientific, Waltham, MA) were conditioned for 1 h in an oven at 250°C, allowed to cool to room temperature, and used immediately for chamber testing. A small amount (100–300 mg) of each oil was placed in an aluminum boat and heated in a micro-chamber/thermal extractor (M-CTE250, Markes International Inc., Gold River, CA) to 25°C (control), 150°C, or 250°C (Figure 1) with sample collection beginning immediately with the first of four tubes as described below. Because of fouling from the oils at high temperatures, the microchamber was cleaned with methylene chloride and baked at 250°C for one h between trials. VEA, vitamin E oil, coconut oil, and MCT were tested. Some of the trials were repeated to check reproducibility of chemical emission profiles: VEA at 250°C, vitamin E at 250°C, coconut oil at 150°C, and MCT at 150°C.

### General VOC Testing

Prior to testing, two background air samples were collected on separate Markes Universal thermal desorption (TD) tubes (Markes International, Inc., Sacramento, CA): one for 20 min and one for 30 s. Prior to placing the oil sample in the microchamber, a background emission sample of a conditioned aluminum foil weigh boat was collected for 30 s. Chemical emissions were collected on four TD tubes every 30 s after placing the room temperature (22°C) oil into the heated chamber. Sampling rate was measured to be 36.0 ± 2.7 mL min^−1^ (mean ± standard deviation) equivalent to ~18 mL of sample collected on each tube.

TD tubes were analyzed using an Ultra-xr autosampler and Unity-xr thermal desorber (Markes International, Inc.) with an Air Toxics cold trap (U-T15ATA-2S, Markes International, Inc.) at a 20:1 split ratio. The general TD parameters included standby split flow of 10 mL min^−1^, flow path temperature at 150°C, and a minimum carrier pressure of 5 psi. The pre-desorption TD parameters included a pre-purge time of 2 min at a split flow of 50 mL min^−1^. The TD tube desorption parameters consisted of a desorb time of 7 min at 280°C with a trap flow of 50 mL min^−1^. The TD trap parameters consisted of a trap purge for 2 min at 50 mL min^−1^ with trap low temperature set at 25°C, followed by a MAX trap heating rate to 290°C, and desorbed for 3 min with a desorb split flow rate of 38 mL min^−1^. The TD system was attached to a GC/MS operated at the same conditions described above for the bulk oil analysis.

### Carbonyl Testing

For carbonyl analysis, samples were collected for VEA heated to 250°C from the microchamber into a 60-mL Teflon® impinger (Savillex, Eden Prairie, MN) containing 5 mL of deionized water at 40 mL min^−1^ for 3 min. Vitamin E oil, MCT, and coconut oil diluents were not tested for carbonyls because VEA was more strongly associated with EVALI than the other oils. Two independent trials were conducted one week apart to check reproducibility of chemical emission testing. After collection, samples were decanted into 16 mL vials, then derivatized with 200 microliters (μL) aqueous 250 mM PFBHA. Vials were left overnight to complete derivatization. The next day, 3 mL of methyl tert-butyl ether (MTBE) was added to each vial. The vial was then shaken for 30 s and allowed to separate into a MTBE layer and aqueous layer. The MTBE was removed and put into a 7-mL vial, blown to dryness, then reconstituted with 100 μL of toluene. The toluene layer was then removed with a pipette and placed in a 2-mL autosampler vial with a 250-μL glass insert (Restek, Bellefonte, PA).

All PFBHA-derivatized samples were analyzed using a Varian (Palo Alto, CA) 3800/Saturn 2000 GC/MS system operated in the EI mode. Compound separation was achieved by an Agilent (Santa Clara, CA) HP-5MS (0.25 mm I.D., 30 m long, 0.25 μm film thickness) column and the following GC oven parameters: 40°C for 2 min., then 5°C min^−1^ to 200°C, then 25°C min^−1^ to 280°C and held for 5 min. One μL of each sample was injected in the splitless mode, and the GC injector was returned to split mode 1 min after sample injection, with the following injector temperature parameters: 130°C for 2 min then 200°C min^−1^ to 300°C and held for 10 min. The Saturn 2,000 ion trap mass spectrometer was tuned using perfluorotributylamine (FC-43). Full-scan EI ionization spectra were collected from *m/*z 40–650.

### Data Analysis

We performed confirmation of identified compounds emitted from heated oils using retention time matching for 34 compounds of interest (Supplementary Table 1) by comparing sample mass spectra to the NIST11 mass spectral library using Masshunter Unknowns Analysis software, manually interrogating the chromatograms using Masshunter Qualitative Analysis Navigator (Agilent Technologies, Inc.), and interrogating the mass spectra for PFBHA loss from derivatized compounds for carbonyls. Carbonyl masses are reported as underivatized mass to charge ratios (*m/z*). Identified compounds are reported for the fourth TD tube (collected beginning at 90 s and ending at 120 s after oil was added to chamber) to allow for temperature equilibration and are reported when the match factor was ≥75% or the compound retention time was matched using an analytical standard. Acetic acid in both VEA 250°C trials was not correctly identified by the automated Masshunter Unknowns Analysis software because of triangular peak shape, overall intensity, and mass spectral noise. It was correctly identified in lower temperature samples (e.g., VEA 150°C). For acetic acid in both VEA 250°C trials, an extracted ion chromatogram for *m/z* 61, 60, 45, 43, and 42 in Masshunter Qualitative Analysis Navigator was used for peak integration between 7.9 and 10.0 min and qualitative match factors. For VOC retention time matching, analytical standards were diluted in methanol or ethanol to ~500 ng/sample and fortified on the front of a TD tube with a 1 μL injection. TD tube spiked samples had nitrogen passed over them for 5 min at 100 mL min^−1^ to push the analytes onto the sorbent beds.

We extracted Globally Harmonized System (GHS) hazard classes (H codes) from PubChem for each identified chemical emission and assigned a hazard group (“–“ < “+” < “++”) with the highest hazard class being used for designation (i.e., a higher hazard group can also include a lower hazard class): “–“ = physical hazard only (H220, H225, H226), or environmental hazard only (H400, H410, H411, H412, H413), or no hazards noted in PubChem; “+” = oral acute toxicity (H301, H302), skin corrosion/irritation (H314, H315, H316), skin sensitization (H317), serious eye damage/eye irritation (H319), respiratory tract irritation or narcotic effects from a single exposure with specific target organ toxicity (H335, H336), germ cell mutagenicity (H340, H341), carcinogenicity (H350, H351), reproductive toxicity (H360D, H360FD, H361, H361d, H361f), and/or repeated exposure with specific target organ toxicity (H372, H373); and “++” = aspiration hazard (H304) and/or acute inhalation toxicity (H330, H331, H332, or H333).

## Results

### Bulk Oil Analysis

For VEA, we confirmed (±)-α-tocopherol acetate as the main constituent of the oil tested, at a retention time of 54.7 min with a small amount of vitamin E. NIST mass spectral matching revealed only a 40% match for VEA but the characteristic *m/z* pattern of 472 (molecular ion), 430, 207, and 165 (base peak) was evident. For vitamin E oil, we confirmed vitamin E as the sole constituent at a retention time of 52.0 min with a NIST mass spectral matching of 32% but the characteristic *m/z* pattern of 430 (molecular ion), 205, and 165 (base peak) were present. For coconut oil and MCT, we identified two main constituents: glycerol tricaprylate (CAS# 538-23-8) at 43.8 min with a 94% match and 2-(decanoyloxy) propane-1,3-diyl dioctanoate (CAS# 33368-87-5) at 51.2 min with an 88% match.

### General VOC Testing

**Figure 2:**
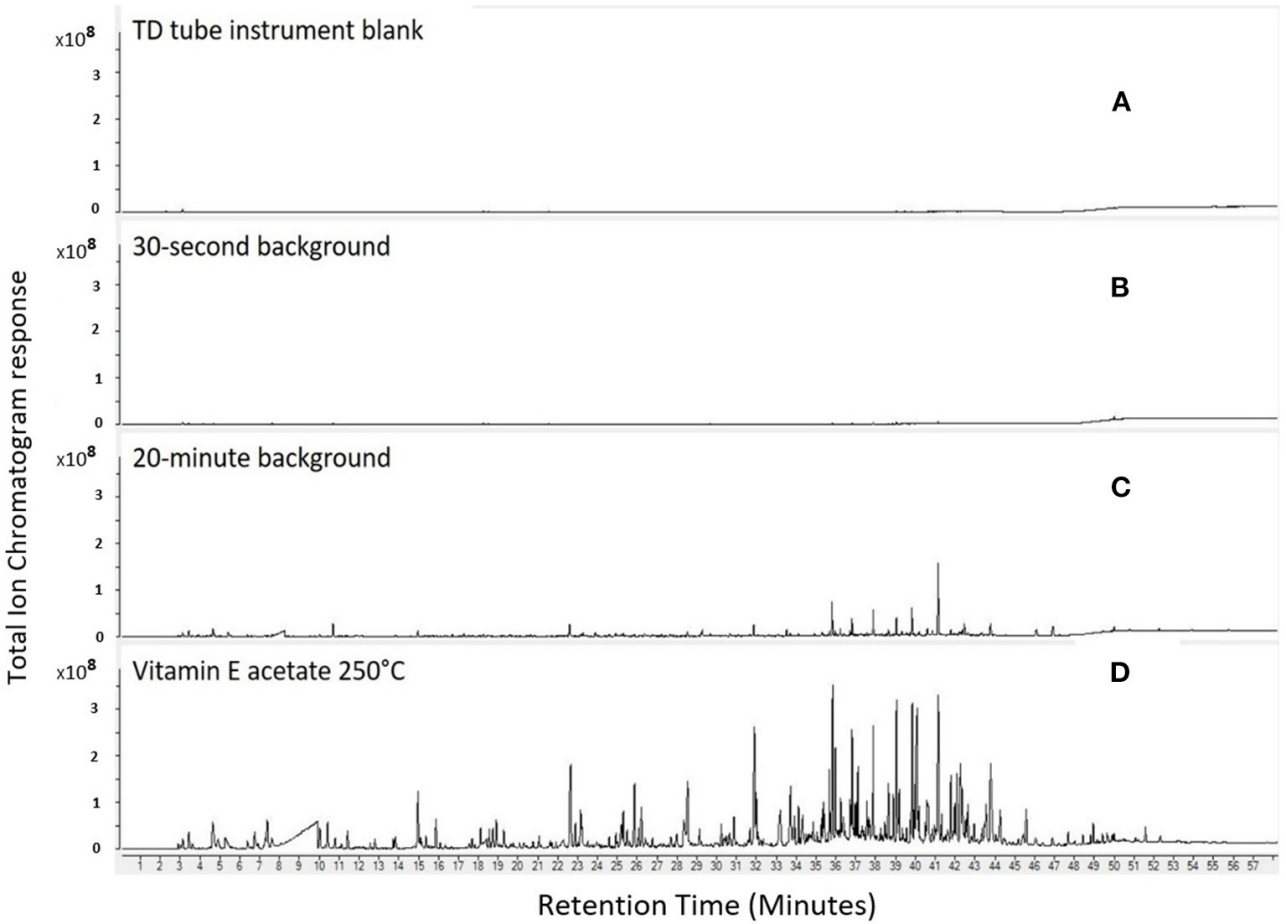
Comparison of TD tube instrument blank **(A)**, 30-s background **(B)**, 20-min background **(C)**, and VEA emissions at 250°C sampled between 90 and 120 s after oil was added to chamber **(D)**.

As expected, the chromatogram for the TD tube instrument blank (Figure 2A) did not show the presence of any VOCs or carbonyls. The background air samples collected for 30 s (Figure 2B) had a few chromatogram peaks at retention times 36–43 min and 48–52 min; however, peaks were minimal in magnitude and number compared to the instrument blank. A second, longer duration (higher volume) background air sample was collected for 20 min (Figure 2C) and the chromatogram contained multiple peaks. Though peaks were present in this 20-min duration blank, their magnitude was much lower compared to the emissions in the chromatogram for VEA heated at 250°C (Figure 2D). These results demonstrated that the chemicals identified in the (30-s and 20-min) background samples did not appreciably influence the emissions identified from the heated VEA samples. For example, acetic acid in the 30-s background accounted for only 0.2% of the integrated area of the total ion chromatogram of acetic acid in the sample collected when VEA was heated at 250°C. Though not shown for brevity, background air samples were similarly low for the tested vitamin E, coconut, and MCT oils.

**Figure 3:**
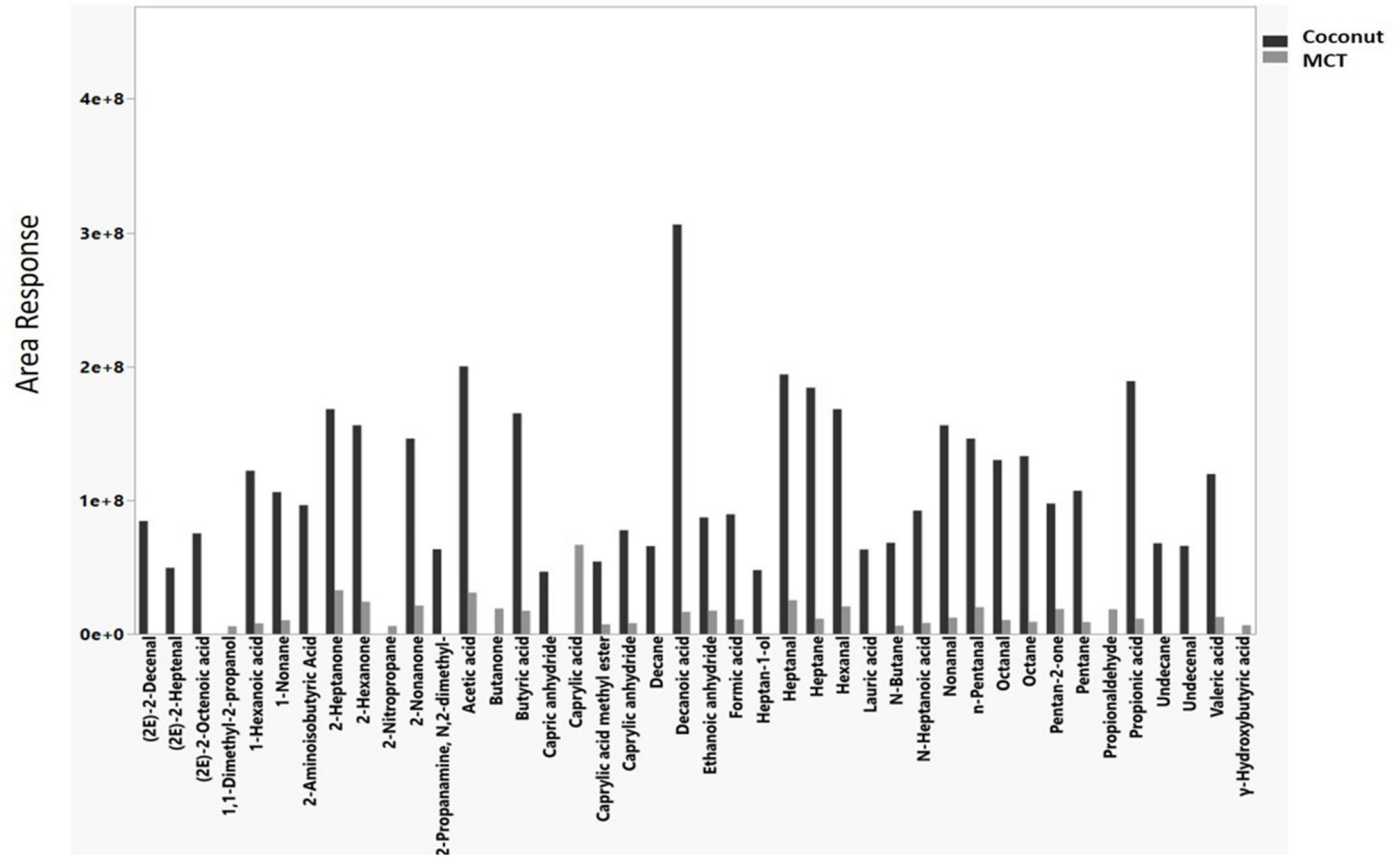
Hazardous chemical emissions from coconut (black bars) and medium chain triglyceride (MCT, gray bars) oil at 250°C during general volatile organic compound sampling.

Chemical emissions confirmed with retention time matching to neat chemical standards as well as hazards associated with those chemicals for heated oil diluents are presented in Table 1. The chemicals elucidated included alcohols, aldehydes, ketones, and saturated and unsaturated hydrocarbons. When VEA was heated in an inert chamber at 250°C in the presence of oxygen (i.e., air flowing over the material), chemical emissions included thermal degradation products such as acetic acid and oxidation products such as 6,10,14-trimethyl-2-pentadecanone (Table 1). Seven hazardous chemical emissions were identified and confirmed from VEA: acetic acid, acetone, formic acid, methylpropenal, isovaleraldehyde, farnesane, and 2-methylheptane. Methylpropenal, farnesane, and 2-methylheptane were not measured during 25°C or 150°C trials. These three chemicals were also categorized into the most hazardous group [i.e., “++,” which is an aspiration hazard (H304) and/or acute inhalation toxicity (H330, H331, H332, or H333)]. One of these hazardous chemicals, 2-Methylheptane, was observed in vitamin E oil emissions at area responses 3–4 times lower than VEA emissions (5.42E+07 compared to 1.60E+08 or 2.22E+08). Acetic acid had the greatest peak area for VEA (2.64E+09 in the first trial and 2.25E+09 in the second trial). Vitamin E oil, coconut, and MCT had some of the same chemical emissions (e.g., acetic acid, formic acid) as VEA but at much lower areas (Table 1). Nonanal and 2-nonanone emissions were unique to coconut oil and MCT (Table 1). Even though coconut and MCT direct analysis of diluted oil revealed the same main constituents, the chemical emissions at 250°C for coconut oil had more hazardous compounds and greater areas (more intense) than MCT (Figure 3). A list of hazards associated with compounds identified in emissions for each oil trial at 250°C can be found in Supplementary Tables 2–8.

**Table 1:** Hazards associated with compounds identified and confirmed by retention time matching in heated emissions of oil diluents at 250°C.

**Oil**	**Trial**	**Common name**	**Formula**	**CAS#**	Hazard^*^	**Match Factor**	**RT**	**Area**
VEA	First	Acetic acid	C2H4O2	64-19-7	+	75	7.7	2.64E+09
VEA	Second	Acetic acid	C2H4O2	64-19-7	+	81	7.7	2.25E+09
VEA	First	Acetone	C3H6O	67-64-1	+	41^a^	4.6	3.97E+08
VEA	Second	Acetone	C3H6O	67-64-1	+	78	4.7	1.60E+08
VEA	First	Formic acid	CH2O2	64-18-6	+	99	5.3	2.11E+08
VEA	Second	Formic acid	CH2O2	64-18-6	+	99	5.3	1.30E+08
VEA	First	Methylpropenal (methacrolein)	C4H6O	78-85-3	++	63	6.7	1.61E+08
VEA	Second	Methylpropenal (methacrolein)	C4H6O	78-85-3	++	97	6.8	1.40E+08
VEA	First	Isovaleraldehyde	C5H10O	590-86-3	+	96	10.1	1.52E+08
VEA	Second	Isovaleraldehyde	C5H10O	590-86-3	+	94	10.1	1.10E+08
VEA	First	2-Methylheptane	C8H18	592-27-8	++	98	15.9	2.22E+08
VEA	Second	2-Methylheptane	C8H18	592-27-8	++	98	15.9	1.60E+08
VEA	First	Farnesane	C15H32	3891-98-3	++	19^a^	35.7	4.24E+08
VEA	Second	Farnesane	C15H32	3891-98-3	++	80	35.7	2.80E+08
VEA	First	Hexahydrofarnesyl acetone^b^	C18H36O	502-69-2	–	81	42.6	2.64E+08
Vitamin E	First	Acetone	C3H6O	67-64-1	+	97	4.9	1.50E+07
Vitamin E	First	Acetic acid	C2H4O2	64-19-7	+	98	8.6	3.24E+07
Vitamin E	Second	Acetic acid	C2H4O2	64-19-7	+	98	8.1	1.02E+08
Vitamin E	Second	2-Methylheptane	C8H18	592-27-8	++	93	15.9	5.42E+07
Vitamin E	Second	Pristane	C19H40	1921-70-6	+	94	39.6	3.13E+08
Vitamin E	First	Hexahydrofarnesyl acetone	C18H36O	502-69-2	–	98	41.5	2.37E+08
Vitamin E	Second	Hexahydrofarnesyl acetone	C18H36O	502-69-2	–	97	41.1	5.05E+08
Vitamin E	Second	Phytol	C20H40O	150-86-7	+	85	42.3	1.57E+08
Coconut	First	Formic acid	CH2O2	64-18-6	+	99	5.4	8.94E+07
Coconut	First	Acetic acid	C2H4O2	64-19-7	+	97	8.4	2.00E+08
Coconut	First	2-Nonanone	C9H18O	821-55-6	+	98	27.4	1.46E+08
Coconut	First	Nonanal	C9H18O	124-19-6	+	99	27.9	1.56E+08
MCT	First	Ethanol	C2H6O	64-17-5	–	99	4.2	4.51E+06
MCT	First	Formic acid	CH2O2	64-18-6	+	98	5.5	1.09E+07
MCT	First	Acetic acid	C2H4O2	64-19-7	+	98	7.9	3.09E+07
MCT	First	2-Nonanone	C9H18O	821-55-6	+	98	27.4	2.12E+07
MCT	First	Nonanal	C9H18O	124-19-6	+	98	27.9	1.22E+07

* *Hazard assigned to groups using PubChem and Globally Harmonized System (GHS) classification hazard class with the highest hazard class being used for designation (i.e., higher hazard group can include lower hazard class): “–“ = Physical hazard only (H220, H225, H226), or environmental hazard only (H400, H410, H411, H412, H413), or no hazards noted in PubChem; “+” = Oral acute toxicity (H301, H302), skin corrosion/irritation (H314, H315, H316), skin sensitization (H317), serious eye damage/eye irritation (H319), respiratory tract irritation or narcotic effects from a single exposure with specific target organ toxicity (H335, H336), germ cell mutagenicity (H340, H341), carcinogenicity (H350, H351), reproductive toxicity (H360D, H360FD, H361, H361d, H361f), and/or repeated exposure with specific target organ toxicity (H372, H373); and “++” = Aspiration hazard (H304) and/or acute inhalation toxicity (H330, H331, H332, or H333)*.

a *These low match factors were a result of noisy mass spectrum compared to NIST11 library*.

b *identified as 1,2-epoxynonadecane, CAS#67860-04-2, in second trial but not confirmed with retention time matching (see Supplementary Table 3). Common name taken from Chemspider (http://www.chemspider.com/). Area is the component area after mass spectral deconvolution. Match factor is the automated NIST mass spectral quality factor ranging from 0 to 100 with higher numbers indicating a better match with standard spectra. RT is the retention time of the deconvoluted peak in minutes*.

**Figure 4:**
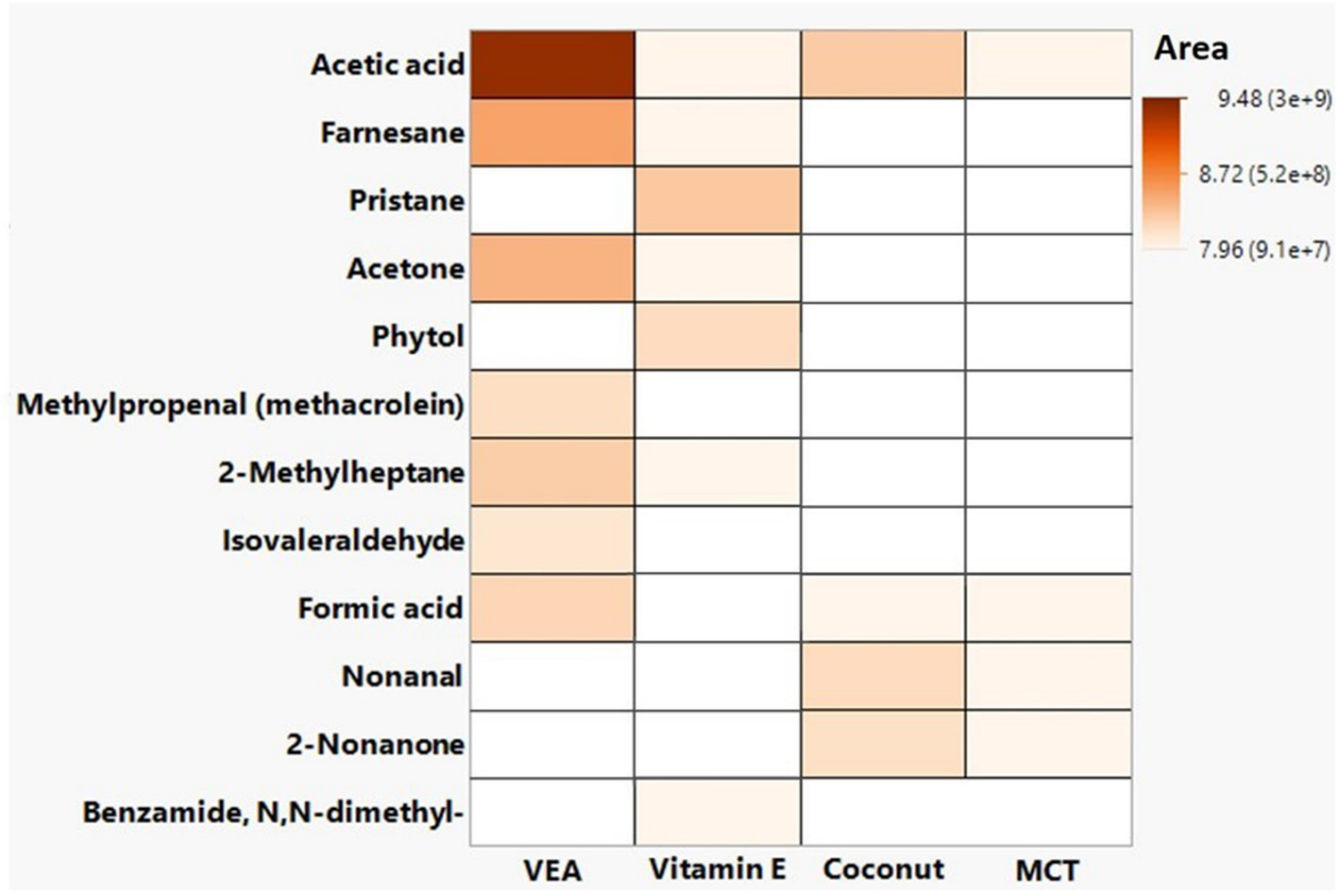
Confirmed hazardous chemical emissions from vitamin E acetate (VEA), vitamin E oil, coconut oil, and medium chain triglycerides (MCT) during general volatile organic compound sampling.

VEA emissions had many chemicals that were unique from the other three oils as can be seen in Figure 4 and Table 1. The unique emissions from heated VEA compared to the other oils included: (1) three chemicals that were identified and confirmed (methylpropenal, isovaleraldehyde, and 6,10,14-Trimethyl-2-pentadecanone); and (2) 30 chemicals that were identified but not confirmed, of which eight have known hazards associated with exposure (2-hexyl-1-octene, 2-ethylcrotonoaldehyde, 3-methylbutanoic acid, 6-methyl-3-heptanol, 3,4-dimethylhex-3-en-2-one, disparlure, dodecan-1-ol, and cis-13-octadecanal).

**Figure 5:**
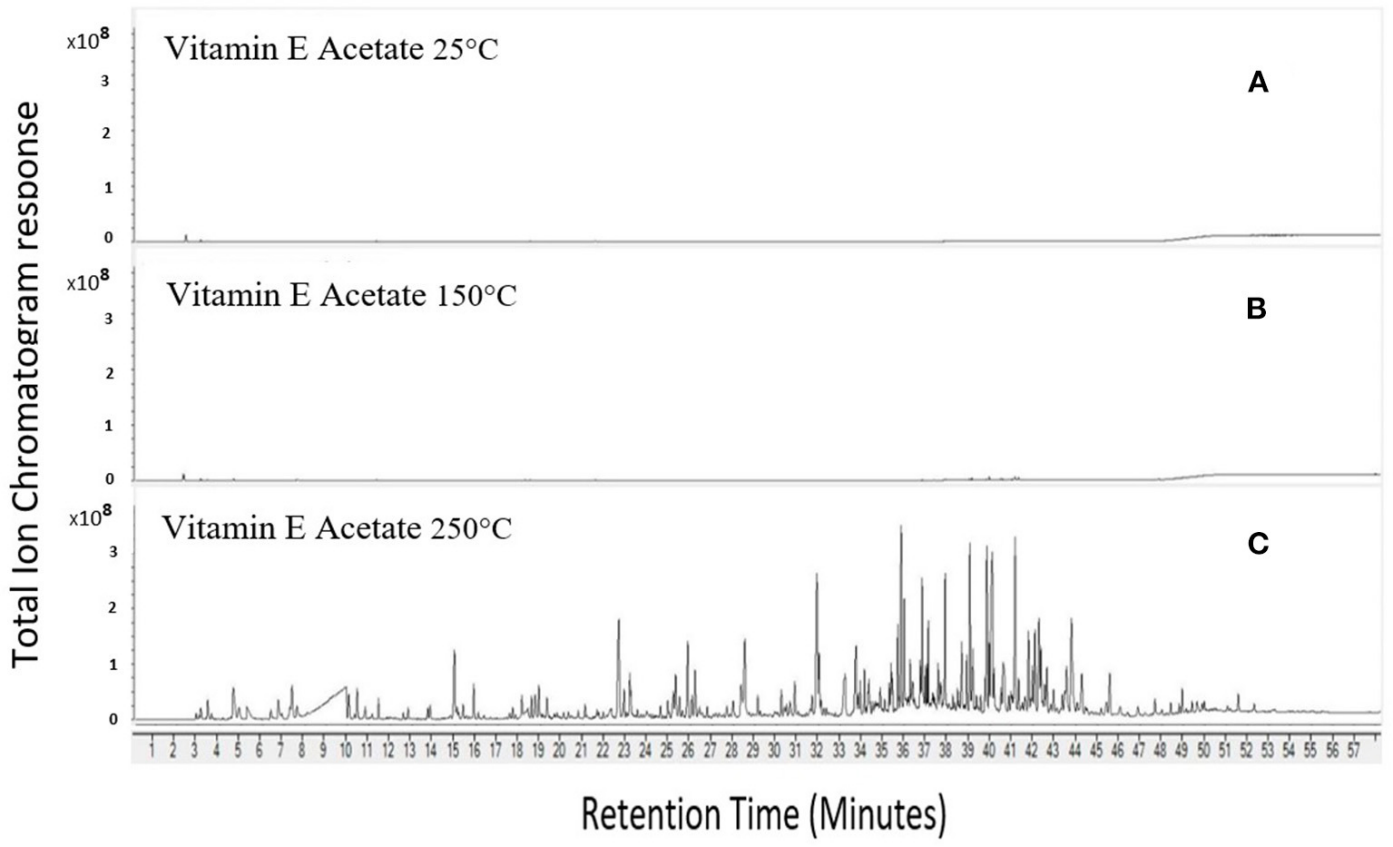
Increasing emissions from VEA with increasing temperature. 25°C **(A)**, 150°C **(B)**, 250°C **(C)**.

**Figure 6:**
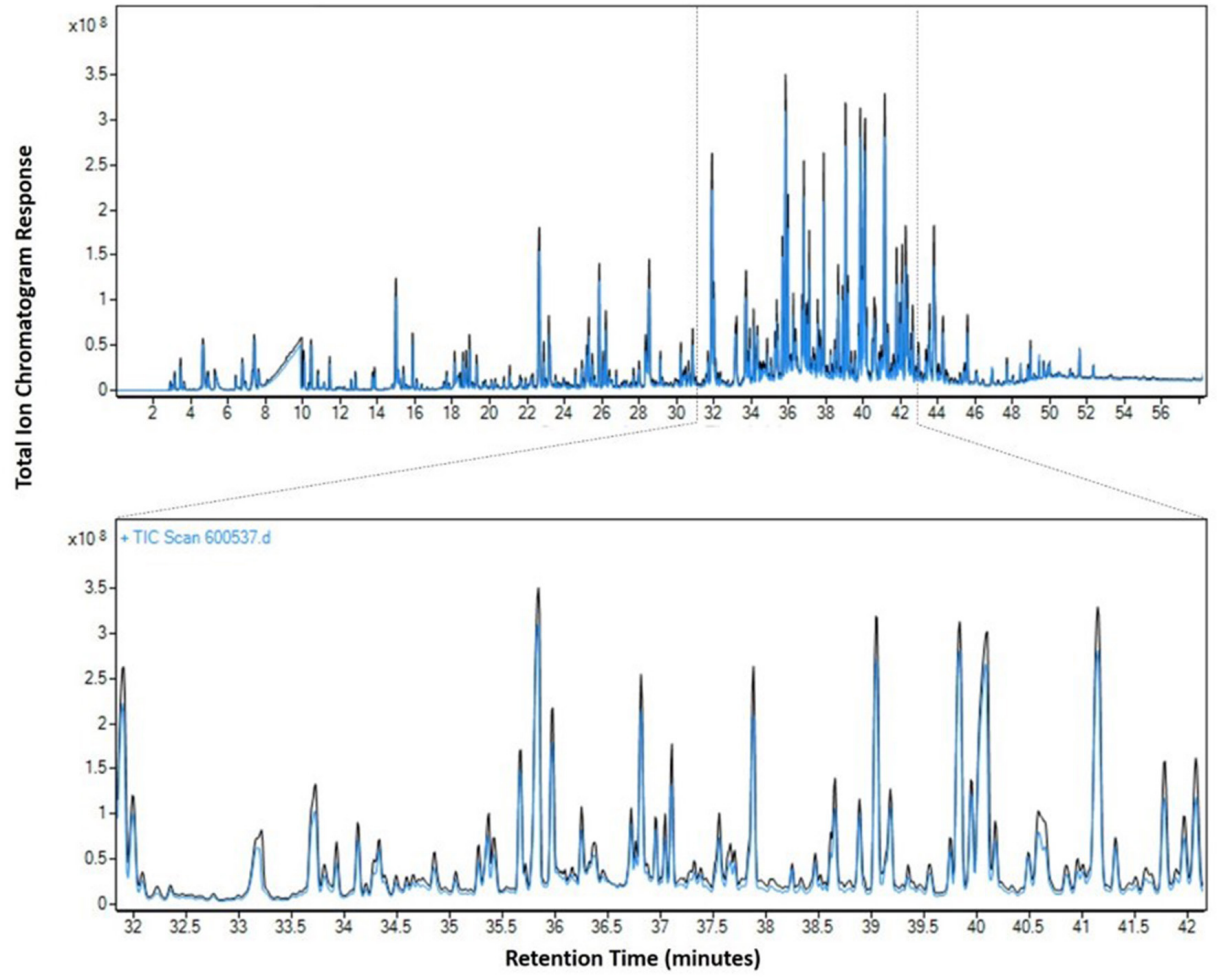
Reproducibility of VEA emissions at 250°C. Blue line = emission test on 11/5/2019 and black line = emission test on 11/14/2019 with a zoomed region from 32 to 42 min.

VEA emissions at 250°C were complex in terms of the number and magnitude of chemicals emitted when compared to 150°C and 25°C (Figure 5). Increasing the temperature from 25°C to 250°C substantially increased emissions from VEA (as evidenced by increased chromatographic peak area response), Acetic acid peak area response was five times more abundant at 150°C compared to 25°C and 418 times more abundant at 250°C compared to 150°C. Acetone peak area increased by ~580 times from 25°C to 250°C. The other six chemicals positively confirmed by retention time matching (6,10,14-trimethyl-2-pentadecanone; formic acid; methylpropenal; isovaleraldehyde; 2-methylheptane; farnesane) were produced at 250°C but not at 25°C or 150°C. VEA emissions at 250°C were reproducible as evidenced by a similar pattern of chemical peaks between replicate tests (Figure 6). On heating VEA at 250°C, we elucidated and confirmed ten hazardous chemicals: acetone, acetic acid, formic acid, methylpropenal, isovaleraldehyde, 2-methylheptane, farnesane, methylglyoxal, diacetyl, and 2-butanone. The latter three chemicals were a direct result of carbonyl testing.

### Carbonyl Testing

**Figure 7:**
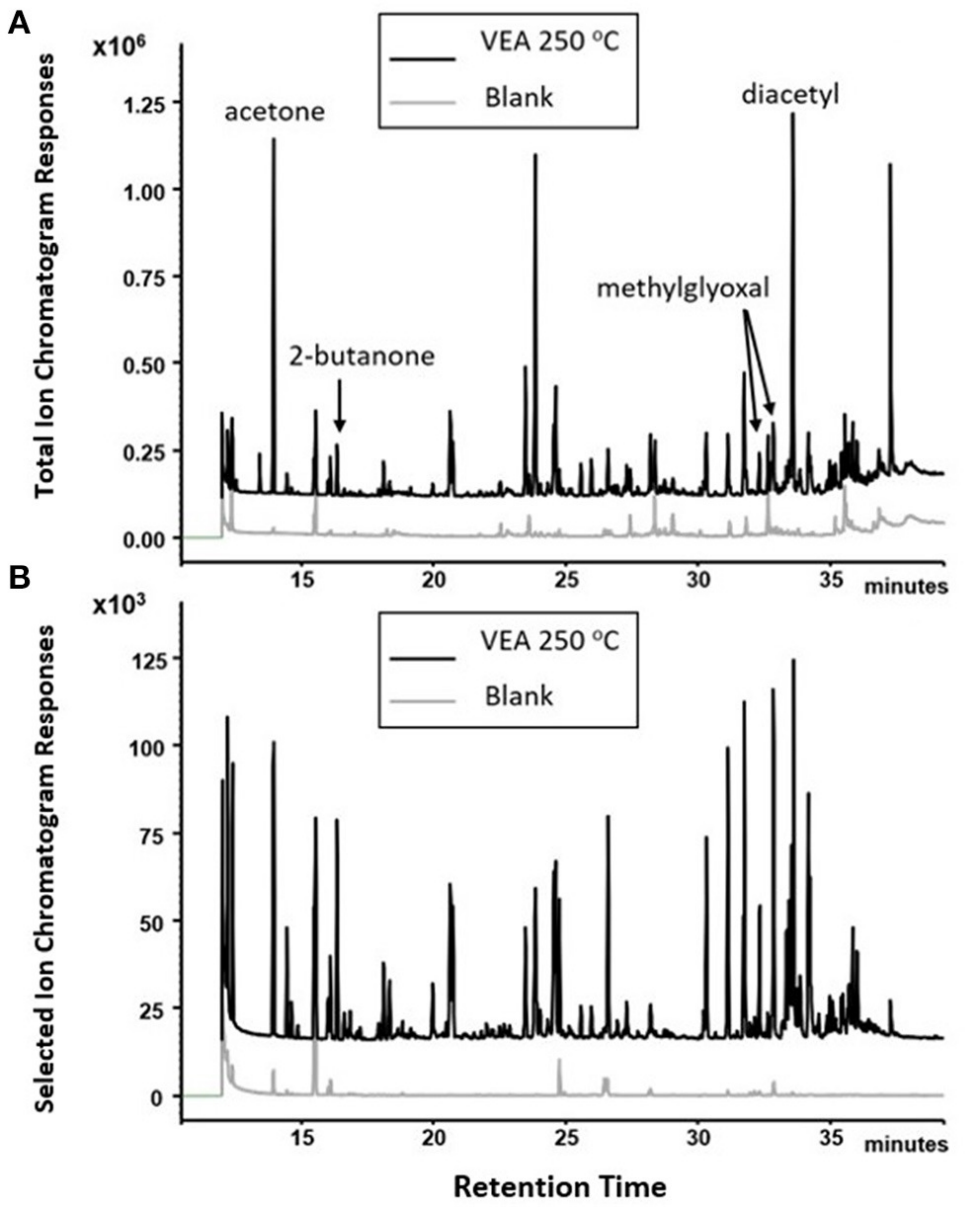
PFBHA derivatization of carbonyls yielded the identification of four chemical emissions of VEA at 250°C: acetone, 2-butanone, methylglyoxal, and diacetyl. **(A)**, total ion current chromatogram; **(B)**, 181.0 ion extracted chromatogram.

As with VOC testing results, VEA emissions at 250°C were complex and several oxygenated chemicals were observed when compared to blank air samples (Figure 7). Although majority of peaks were not identified in PFBHA-derivatized samples, four carbonyl compounds were elucidated using neat standards: acetone (13.9 min), 2-butanone (16.1 and 16.3 min), methylglyoxal (32.3 and 32.8 min), and diacetyl (33.6 min). Other major carbonyl peaks observed were at 20.6 and 20.7 min (*m/z* = 100), 23.5 and 23.8 min (*m/z* = 128), 24.5 and 24.6 min (*m/z* = 98), and 37.3 min (*m/z* = 222). Using PFBHA as a derivatization agent and subsequently extracting the 181 ion from mass spectra (Figure 7B), aids in identifying that peaks observed were attributable to carbonyl species.

Acetone, 2-butanone, methylglyoxal, and diacetyl were not observed in blank air samples collected from micro-chamber, which indicated that they were generated from oxidation of VEA at 250°C. Additionally, the other major carbonyl species were only observed when VEA was present and heated.

## Discussion

In this study, diluent oils were heated to temperatures consistent with wet-through-wick (145–334°C) and full-wet conditions (110–185°C) (11). The temperature at the center of a coil can be up to 100°C hotter than the sides (15). THC-extract oil should be heated between 220 and 230°C to maximize aerosolization and minimize combustion (16). Diluent oils heated to temperatures representative of use in EVPs emitted hazardous chemicals that were a result of volatilization, thermal degradation, or reactions (oxidation or rearrangements). The elucidated chemicals included alcohols, aldehydes, ketones, and saturated and unsaturated hydrocarbons, that when inhaled simultaneously together might have deleterious effects locally on the respiratory system or systemically. We positively identified seven hazardous chemicals emitted from VEA when heated to 250°C that were not present at 150°C, and the emissions were reproducible, although the exposure time to that temperature was longer than typical puff durations (~3.5 ± 1.4 s) observed for nicotine-containing e-cigarettes (17).

Extended temperature exposure times used in this study (i.e., 30 s) could change the heated chemical emissions compared to shorter puff durations typically employed by users. However, several other researchers have observed heated VEA emissions similar to our results. Riordan-Short et al. identified formic acid, acetone, isovaleraldehyde, acetic acid, and methylpropenal (i.e., methacrolein) in the headspace of heated VEA at 300°C (18). Jiang et al. observed thermal decomposition and oxidation product formation including carbonyls from aerosolization of oil diluents using a commercially available vape pen (19). Similar to this study, they observed acetone emissions from vitamin E oil and VEA as well as butanone, n-pentanal, 2-pentanone, and 2-heptanone from MCT oil. Mikheev et al. observed duroquinone and 1-pristene, similar to Wu and Shea (13) but also durohydroquinone monoacetate, from direct extraction of vaped VEA condensate using methylene chloride with no derivatization (20). Lynch et al. observed duroquinone, 1-pristene, and durohydroquinone monoacetate in vaped THC/VEA aerosol condensate from commercially available ceramic coil vaping cartridges (12). They used an elevated power setting of 16 W corresponding to 415–476°C at 5 V with temperatures exceeding those used in this study and measured derivatized condensate instead of measuring gas-phase native species of heated emissions as in this study. Direct comparisons to studies of vaped emission condensate that are native or derivatized and collected at different temperatures should be made with caution but data from our study complements information obtained elsewhere. Although we did not observe duroquinone or durohydroquinone in this study, we did observe quinone-containing compounds from vitamin E oil (Supplementary Table 5) but not from VEA. Of note, hexahydrofarnesyl acetone, C18H36O, CAS# 502-69-2 observed in this study is an oxidized form of, and structurally similar to, 1-pristene, a degradation product of VEA, observed elsewhere (12, 20).

Hazards associated with these emitted chemicals included aspiration hazards and/or acute inhalation toxicity. While aspiration hazards can cause acute effects in the lungs, in the context of EVALI, early reports suggested exogenous lipoid pneumonia from aspiration of e-liquids, though subsequently it was shown that this pathology was not consistent with EVALI (21, 22). Rather, lung biopsies of EVALI cases indicated acute lung injury, including organizing pneumonia and/or diffuse alveolar damage, which suggested that inhalation of aerosols is the more likely cause of lung damage (21, 22). The importance of the inhalation pathway in EVALI highlights the importance of understanding the chemistry of emissions from oil diluents. VEA is associated with EVALI (2, 3), though whether the effects are from aerosolized VEA, its gas-phase thermal degradation products, or a combined effect is unclear. In this study, heating VEA to a temperature consistent with user device settings emitted a myriad of gas-phase chemicals (alcohols, aldehydes, ketones, and saturated and unsaturated hydrocarbons) associated with adverse respiratory health effects. None of these compounds individually yield the same pathology observed with EVALI, though whether the combined effects on the respiratory system could contribute to EVALI remains unclear and warrants further investigation. The findings of relatively high emissions of acetone and acetic acid are significant given both are known precursors for ethenone (23). These gas-phase emissions can deposit along the respiratory tract and reach the deep lung (i.e., small airways) depending on partitioning behavior of the chemicals. A portion of these gas-phase compounds will remain partitioned in aerosolized liquid droplets (24) produced by heating VEA. These liquid droplets will deposit in the respiratory tract, and as described in our companion paper, have sub-micron scale, and will therefore predominantly reach the lung alveoli, which is the site of injury of EVALI (25).

Dicarbonyls such as methylglyoxal and diacetyl have been shown to cause adverse respiratory health effects (26). Exposure to methylglyoxal and diacetyl can induce necrosis in lung epithelial cells (26). Furthermore, exposure to diacetyl can induce development of bronchiolitis obliterans, a severe respiratory illness characterized by damage to the small airways proximal to the alveoli (26, 27). In the current study, diacetyl was generated as an emission product when VEA was heated to 250°C, but we did not measure concentrations making it difficult to say whether the concentrations were high enough to invoke damage. The pathology of bronchiolitis obliterans appears to differ from the pathology of EVALI, the latter which is thought to be chemical pneumonitis, that for most patients, manifested as damage in the alveoli (21, 22, 28). Some EVALI cases with diffuse alveolar damage and organizing pneumonia also had evidence of bronchiolitis (21, 28), but it is unclear if this pathology indicates a possible role for diacetyl.

Given the increase in marijuana use and legalization across the United States, adverse respiratory health effects associated with inhalation of aerosolized oils could continue to be an issue. VEA has been used to dilute THC oil and has been identified in a high proportion (94%) of bronchoalveolar lavage fluid samples from EVALI cases (29). Separate from the recent outbreak in EVALI, a severe case of bronchiolitis has been observed in a Canadian youth who aerosolized THC oil and flavorings, which highlights the need to understand the influence of flavoring components of these complex mixtures (30).

This study had several limitations. First, VEA was heated in an aluminum dish within a heated chamber instead of an aerosolization device designed for inhalation of THC-containing products. Attfield et al. dissected cartridges used for aerosolizing THC-containing products and analyzed the materials chemistry (23). The authors noted that the cartridges contained nickel and chromium coils that were encased in charred oil-soaked, silica ceramic, which is favorable to ethenone formation (but not present in aluminum dishes used in the current study). Consequently, any effects of aerosolized products derived from the device and components used in the devices to aerosolize THC-containing products were not mimicked by our protocol. Second, Lynch et al. observed that unknowns analysis software can incorrectly identify chemicals (12). We attempted to manually investigate the chromatograms to identify compounds but were sometimes unsuccessful because of noisy mass spectra or coeluting peaks. These identities have been assigned based on a mass spectral deconvolution and matching software that might have been influenced by noisy mass spectra from column bleed or by detector saturation. In addition, the TD tube sampling technique did not capture reactive species such as the proposed formation of ketene from pyrolysis of VEA (13). Finally, our work focused only on individual oils used for dilution of THC-containing products and did not examine other THC-based mixtures believed to be involved in EVALI. Muthumalage et al. (10) evaluated a counterfit vape cartridge and reported a significant increase in toxicity from the aerosolized mixture compared with MCT or VEA alone.

## Conclusion

VEA and other oil diluents emit a wide variety of hazardous chemicals when heated to a temperature mimicking electronic delivery devices for THC-aerosolization. Numerous evolved compounds are known respiratory irritants, acute toxins, and known precursors to highly reactive ketenes. This information provides novel insights for investigators to study chemical toxicity in concert with aerosol deposition modeling to better understand the mechanism(s) of EVALI. This data further supports current Centers for Disease Control and Prevention (CDC) guidance to not add VEA or any other substances not intended by the manufacturer to the vaping products (1).

## Data Availability Statement

The original contributions presented in the study are included in the article/Supplementary Material, further inquiries can be directed to the corresponding author/s.

## Author Contributions

RL and AS contributed to the conception of the study. RL, JH, and AS wrote the first draft of the manuscript. MA, KW, EF, and JH collected and analyzed the samples. DB and AR consulted on data collection and analysis. All authors contributed to the article and approved the submitted version.

## Author Disclaimer

The findings and conclusions in this report are those of the authors and do not necessarily represent the official position of the National Institute for Occupational Safety and Health, Centers for Disease Control and Prevention. In addition, citations to websites external to NIOSH do not constitute NIOSH endorsement of the sponsoring organizations or their programs or products. Furthermore, NIOSH is not responsible for the content of these websites. All web addresses referenced in this document were accessible as of the publication date.

## Conflict of Interest

The authors declare that the research was conducted in the absence of any commercial or financial relationships that could be construed as a potential conflict of interest.

## Publisher's Note

All claims expressed in this article are solely those of the authors and do not necessarily represent those of their affiliated organizations, or those of the publisher, the editors and the reviewers. Any product that may be evaluated in this article, or claim that may be made by its manufacturer, is not guaranteed or endorsed by the publisher.
