# Supplementary material for: Chemical Emissions From Heated Vitamin E Acetate—Insights to Respiratory Risks From Electronic Cigarette Liquid Oil Diluents Used in the Aerosolization of Δ9-THC-Containing Products
Source: Front Public Health. 2022 Jan 21;9:765168. doi: 10.3389/fpubh.2021.765168 (PMC8814346; doi:10.3389/fpubh.2021.765168)
Supplement: Supplementary file 1 [file Data_Sheet_1.PDF]

Supplementary Table S1. Retention times for neat chemical standards used for confirmation of chemicals identified in heated oil emissions

Common Name	CAS#	Retention Time (RT) in minutes
Methanol	67-56-1	3.4
Ethanol	64-17-5	4.4
Acetone	67-64-1	4.9
Formic acid	64-18-6	5.2
Methylpropenal	78-85-3	6.8
Vinyl acetate	108-05-4	7.4
Acetic acid	64-19-7	7.7
Isovaleraldehyde	590-86-3	10.0
Ethanoic anhydride	108-24-7	12.6
2-Methylheptane	592-27-8	15.9
(R)-(+)-1,2-Epoxyhexane	1436-34-6	16.8
2-Ethyl-2-butenal	19780-25-7	17.1
Acetic acid, 2-methoxy-	625-45-6	17.7
Hexamethylcyclotrisiloxane	541-05-9	18.3
2-Methylpentyl formate	381670-34-4	20.3
3-Methyl-1-(3-methylbutoxy)butane	544-01-4	24.9
1-Ethyl-2-pyrrolidinone	2687-91-4	27.2
2-Nonanone	821-55-6	27.4
Nonanal	124-19-6	27.9
1-Decanal	112-31-2	31.1
Benzamide, N,N-dimethyl-	611-74-5	35.0
2-Hexyl-1-octene	19780-80-4	35.3
Farnesane	3891-98-3	35.7
1-Octanol, 2-butyl-	3913-02-8	35.8
Undecylic acid	112-37-8	36.7
Diethyl phthalate	84-66-2	37.9
Pristane	1921-70-6	39.6
1-Hexadecanal	629-80-1	40.7
Hexahydrofarnesyl acetone	502-69-2	41.1

Common Name	CAS#	Retention Time (RT) in minutes
1-Eicosene	3452-07-1	43.7
(+)-Disparlure	29804-22-6	44.3
2-Hexadecyloxirane	7390-81-0	44.3
Phytol	150-86-7	46.2
Tricaprilin	538-23-8	47.9

Supplementary Table S2. Vitamin E acetate, first trial: Hazards associated with compounds identified in heated emissions at 250°C

Common Name	Formula	CAS#	Hazard*	Match Factor	RT	RT Match	Area	%total area
Acetone	C3H6O	67-64-1	+	41[a]	4.6	Yes	3.97E+08	2.6
Formic acid	CH2O2	64-18-6	+	99	5.3	Yes	2.11E+08	1.4
Methylpropenal (methacrolein)	C4H6O	78-85-3	++	63	6.7	Yes	1.61E+08	1.1
Butyloxirane	C6H12O	1436-34-6	+	81	7.4	No	2.11E+08	1.4
Pivalolactone	C5H8O2	1955-45-9	–	80	7.4	Not tested	1.55E+08	1.0
Acetic acid	C2H4O2	64-19-7	+	75	7.7	Yes	2.64E+09	17.3
Ethanoic anhydride	C4H6O3	108-24-7	++	81	9.9	No	4.53E+08	3.0
Isovaleraldehyde	C5H10O	590-86-3	+	96	10.0	Yes	1.52E+08	1.0
2-Ethylcrotonaldehyde	C6H10O	19780-25-7	+	94	10.4	Not tested	2.18E+08	1.4
2-Methylheptane	C8H18	592-27-8	++	98	15.9	Yes	2.22E+08	1.5
3-Methylbutanoic acid	C5H10O2	503-74-2	+	92	18.4	Not tested	2.16E+08	1.4
3-Heptanol, 6-methyl-	C8H18O	18720-66-6	+	87	18.7	Not tested	1.83E+08	1.2
3,4-Dimethylhex-3-en-2-one	C8H14O	1635-02-5	+	83	18.9	Not tested	1.52E+08	1.0
(3E)-3-Methyl-3-hepten-2-one	C8H14O	39899-08-6	–	79	18.9	Not tested	1.48E+08	1.0
6-Methyl-2-heptanol, trifluoroacetate	C10H17F3O2	1000365-05-8	–	84	23.2	Not tested	2.00E+08	1.3
2,5-Pyrrolidinedione, 3-methyl-3-propyl-	C8H13NO2	1497-19-4	–	77	25.9	Not tested	2.37E+08	1.6
2,3,7-Trimethyloctane	C11H24	62016-34-6	–	84	28.3	Not tested	3.62E+08	2.4
2-Undecanone	C11H22O	112-12-9	–	81	30.9	Not tested	2.02E+08	1.3
1-Ethyl-2-pyrrolidinone	C6H11NO	2687-91-4	+	78	32.0	No	2.71E+08	1.8
N-(2-Methyl-2-propen-1-yl)hexanamide	C10H19NO	1000340-14-0	–	77	32.0	Not tested	2.53E+08	1.7
2-Ethyl-1,1-dimethylcyclopentane	C9H18	54549-80-3	–	85	33.7	Not tested	3.54E+08	2.3
2-Hexyl-1-octene	C14H28	19780-80-4	++	93	34.1	Not tested	1.99E+08	1.3
Undecylic acid	C11H22O2	112-37-8	+	82	35.4	No	2.28E+08	1.5
Farnesane	C15H32	3891-98-3	++	19[a]	35.7	Yes	4.24E+08	2.8
2-Methyl-1-decanol	C11H24O	18675-24-6	–	89	36.0	Not tested	4.93E+08	3.2
2-Butyl-1-octanol	C12H26O	3913-02-8	–	90	36.7	No	2.06E+08	1.4
Disparlure	C19H38O	29804-22-6	+	77	36.8	Not tested	4.42E+08	2.9
6-Methyl-2-tridecanone	C14H28O	73105-73-4	–	86	37.1	Not tested	2.91E+08	1.9

Common Name	Formula	CAS#	Hazard*	Match Factor	RT	RT Match	Area	%total area
Hexahydrofarnesol	C15H32O	6750-34-1	–	83	37.6	Not tested	1.93E+08	1.3
Dodecan-1-ol	C12H26O	112-53-8	+	83	37.6	Not tested	2.01E+08	1.3
2-Butyl-1,1,3-trimethylcyclohexane	C13H26	54676-39-0	–	81	37.9	Not tested	5.36E+08	3.5
Hexahydrofarnesol	C15H32O	6750-34-1	–	92	38.7	Not tested	2.89E+08	1.9
Hexadecanal	C16H32O	629-80-1	+	75	39.1	No	6.92E+08	4.5
Diisoamyl ether	C10H22O	544-01-4	++	75	39.8	No	2.17E+08	1.4
Z-5-Nonadecene	C19H38	1000131-11-8	–	86	40.0	Not tested	2.93E+08	1.9
(3E,6E)-1,3,6-Octatriene	C8H12	929-20-4	–	79	40.1	Not tested	4.37E+08	2.9
2,10-Dimethyl-9-undecenal	C13H24O	1000131-85-9	–	84	41.8	Not tested	4.04E+08	2.7
Disparlure	C19H38O	29804-22-6	+	75	42.3	Not tested	5.70E+08	3.7
Hexadecenal	C16H30O	22644-96-8	–	84	42.4	Not tested	3.53E+08	2.3
Hexahydrofarnesyl acetone	C18H36O	502-69-2	–	81	42.6	Yes	2.64E+08	1.7
2-Hexadecyloxirane	C18H36O	7390-81-0	+	89	43.6	No	3.35E+08	2.2
cis-13-Octadecenal	C18H34O	58594-45-9	+	76	43.8	Not tested	7.84E+08	5.1

*Hazard assigned to groups using PubChem and Globally Harmonized System (GHS) classification hazard class with the highest hazard class being used for designation (i.e., higher hazard group can include lower hazard class):

"–" = Physical hazard only (H220, H225, H226), or environmental hazard only (H400, H410, H411, H412, H413), or no hazards noted in PubChem;

"+" = Oral acute toxicity (H301, H302), skin corrosion/irritation (H314, H315, H316), skin sensitization (H317), serious eye damage/eye irritation (H319), respiratory tract irritation or narcotic effects from a single exposure with specific target organ toxicity (H335, H336), germ cell mutagenicity (H340, H341), carcinogenicity (H350, H351), reproductive toxicity (H360D, H360FD, H361, H361d, H361f), and/or repeated exposure with specific target organ toxicity (H372, H373); and

"++" = Aspiration hazard (H304) and/or acute inhalation toxicity (H330, H331, H332, or H333).

[a]low match factors were a result of noisy mass spectrum compared to NIST11 library.

Notes: Common name taken from Chemspider (http://www.chemspider.com/). Area is the component area after mass spectral deconvolution. %Area is the percentage of reported component area (i.e., sum of %Area equals 100%). Match factor is the automated NIST mass spectral quality factor ranging from 0 to 100 with higher numbers indicating a better match with standard spectra. Some chemicals are repeated at different retention times indicating incompatibility of the chemical with the column leading to multiple peaks, geometric isomers, or inappropriate mass spectral identification.

Supplementary Table S3. Vitamin E acetate, second trial: Hazards associated with compounds identified in heated emissions at 250°C

Common Name	Formula	CAS#	Hazard*	Match Factor	RT	RT match	Area	%total area
Acetone	C3H6O	67-64-1	+	78	4.7	Yes	1.6E+08	1.2
Formic acid	CH2O2	64-18-6	+	99	5.3	Yes	1.3E+08	1.0
Methylpropenal (methacrolein)	C4H6O	78-85-3	++	97	6.8	Yes	1.4E+08	1.0
Acetic acid	C2H4O2	64-19-7	+	81	7.7	Yes	2.3E+09	16.9
Ethanoic anhydride	C4H6O3	108-24-7	++	87	9.9	No	8.4E+08	6.3
Isovaleraldehyde	C5H10O	590-86-3	+	94	10.1	Yes	1.1E+08	0.9
2-Ethylcrotonaldehyde	C6H10O	19780-25-7	+	94	10.5	Not tested	1.9E+08	1.4
Butyloxirane	C6H12O	1436-34-6	+	82	15.0	No	2.5E+08	1.9
2-Methylheptane	C8H18	592-27-8	++	98	15.9	Yes	1.6E+08	1.2
1-(3-Ethylcyclobutyl)ethanone	C8H14O	56335-71-8	–	82	18.9	Not tested	1.4E+08	1.0
6-Methyl-2-heptanol, trifluoroacetate	C10H17F3O2	1000365-05-8	–	83	23.2	Not tested	1.4E+08	1.0
2,5-Pyrrolidinedione, 3-methyl-3-propyl-	C8H13NO2	1497-19-4	–	77	25.9	Not tested	2.0E+08	1.5
2-Nonanone	C9H18O	821-55-6	+	85	26.2	No	2.2E+08	1.7
2,3,7-Trimethyloctane	C11H24	62016-34-6	–	84	28.3	Not tested	2.4E+08	1.8
2-Methylpentyl formate	C7H14O2	381670-34-4	+	81	28.5	No	3.3E+08	2.5
Decyl prop-1-en-2-yl carbonate	C14H26O3	1000382-90-5	–	83	30.9	Not tested	1.5E+08	1.2
1-Ethyl-2-pyrrolidinone	C6H11NO	2687-91-4	+	78	32.0	No	1.8E+08	1.4
N-(2-Methyl-2-propen-1-yl)hexanamide	C10H19NO	1000340-14-0	–	78	32.0	Not tested	2.3E+08	1.7
2-Ethyl-1,1-dimethylcyclopentane	C9H18	54549-80-3	–	85	33.7	Not tested	2.8E+08	2.1
2-Hexyl-1-octene	C14H28	19780-80-4	++	94	34.1	Not tested	1.6E+08	1.2
Undecylic acid	C11H22O2	112-37-8	+	79	35.4	No	1.4E+08	1.1
Farnesane	C15H32	3891-98-3	++	80	35.7	Yes	2.8E+08	2.1
6,10,14-Trimethyl-2-pentadecanol	C18H38O	69729-17-5	–	89	36.0	Not tested	4.0E+08	3.0
2-(Diethylamino)butanenitrile	C8H16N2	16250-35-4	–	79	36.0	Not tested	3.1E+08	2.3
2-Butyl-1-octanol	C12H26O	3913-02-8	–	90	36.7	No	1.5E+08	1.1
Disparlure	C19H38O	29804-22-6	+	76	36.8	No	3.7E+08	2.8
6-Methyl-2-tridecanone	C14H28O	73105-73-4	–	86	37.1	Not tested	2.0E+08	1.5
2-Butyl-1,1,3-trimethylcyclohexane	C13H26	54676-39-0	–	81	37.9	Not tested	4.3E+08	3.2

Common Name	Formula	CAS#	Hazard*	Match Factor	RT	RT match	Area	%total area
Hexahydrofarnesol	C15H32O	6750-34-1	–	93	38.7	Not tested	2.1E+08	1.6
2-Hexadecyloxirane	C18H36O	7390-81-0	+	83	39.1	No	6.6E+08	5.0
2-Hydroxy-3,4-dimethyl-2-cyclopenen-1-one	C7H10O2	21835-00-7	–	75	39.8	Not tested	2.9E+08	2.2
Z-5-Nonadecene	C19H38	1000131-11-8	–	86	40.0	Not tested	2.8E+08	2.1
Cyclopropylidenecyclopentane	C8H12	14949-48-5	–	76	40.1	Not tested	3.9E+08	2.9
Hexahydrofarnesyl acetone	C18H36O	502-69-2	–	84	41.2	Not tested	8.8E+08	6.6
2,10-Dimethyl-9-undecenal	C13H24O	1000131-85-9	–	84	41.8	Not tested	2.8E+08	2.1
5-Methyl-5-(4,8,12-trimethyltridecyl)dihydro-2(3H)-furanone	C21H40O2	96168-15-9	–	87	42.0	Not tested	1.8E+08	1.4
Octyldodecanol	C20H42O	5333-42-6	–	78	42.1	Not tested	3.0E+08	2.2
Hexadecenal	C16H30O	22644-96-8	–	84	42.4	Not tested	2.4E+08	1.8
1,2-Epoxynonadecane	C19H38O	67860-04-2	–	82	42.7	Not tested	1.7E+08	1.3
Phytol	C20H40O	150-86-7	+	83	43.8	Not tested	6.6E+08	4.9

*Hazard assigned to groups using PubChem and Globally Harmonized System (GHS) classification hazard class with the highest hazard class being used for designation (i.e., higher hazard group can include lower hazard class):

"–" = Physical hazard only (H220, H225, H226), or environmental hazard only (H400, H410, H411, H412, H413), or no hazards noted in PubChem;

"+" = Oral acute toxicity (H301, H302), skin corrosion/irritation (H314, H315, H316), skin sensitization (H317), serious eye damage/eye irritation (H319), respiratory tract irritation or narcotic effects from a single exposure with specific target organ toxicity (H335, H336), germ cell mutagenicity (H340, H341), carcinogenicity (H350, H351), reproductive toxicity (H360D, H360FD, H361, H361d, H361f), and/or repeated exposure with specific target organ toxicity (H372, H373); and

"++" = Aspiration hazard (H304) and/or acute inhalation toxicity (H330, H331, H332, or H333).

Notes: Common name taken from Chemspider (http://www.chemspider.com/). Area is the component area after mass spectral deconvolution. %Area is the percentage of reported component area (i.e., sum of %Area equals 100%). Match factor is the automated NIST mass spectral quality factor ranging from 0 to 100 with higher numbers indicating a better match with standard spectra. RT is the retention time of the deconvoluted peak.

Supplementary Table S4. Vitamin E oil, first trial: Hazards associated with compounds identified in heated emissions at 250°C

Common Name	Formula	CAS#	Hazard*	Match Factor	RT	RT Match	Area	%total area
Trimethylamine	C3H9N	75-50-3	++	98	4.2	Not tested	1.78E+07	0.8
Acetone	C3H6O	67-64-1	+	97	4.9	Yes	1.50E+07	0.7
Isohexane	C6H14	107-83-5	+	97	7.7	Not tested	1.34E+07	0.6
Acetic acid	C2H4O2	64-19-7	+	98	8.6	Yes	3.24E+07	1.4
Dimethylformamide	C3H7NO	68-12-2	++	98	15.0	Not tested	5.08E+07	2.3
3-Ethylbutanal	C6H12O	15877-57-3	+	91	15.3	Not tested	1.53E+07	0.7
Toluene	C7H8	108-88-3	++	93	15.7	Not tested	2.30E+07	1.0
2-Methylheptane	C8H18	592-27-8	++	97	16.2	Not tested	1.47E+07	0.7
Benzaldehyde	C7H6O	100-52-7	+	97	22.9	Not tested	2.23E+07	1.0
2-Isooctanone	C8H16O	928-68-7	+	95	22.9	Not tested	4.51E+07	2.0
2-Heptanol, 6-methyl-	C8H18O	4730-22-7	–	96	23.5	Not tested	1.11E+07	0.5
2,3-Dimethylmaleic anhydride	C6H6O3	766-39-2	+	97	24.9	Not tested	2.20E+07	1.0
4-Methyldecane	C11H24	2847-72-5	–	96	26.2	Not tested	4.16E+07	1.9
Decamethylcyclopentasiloxane	C10H30O5Si5	541-02-6	+	85	30.6	Not tested	1.17E+07	0.5
2,6-Dimethylundecane	C13H28	17301-23-4	–	95	32.3	Not tested	3.61E+07	1.6
1-(4-Bromobutyl)-2-piperidinone	C9H16BrNO	195194-80-0	–	84	33.6	Not tested	2.39E+07	1.1
6,6-Dimethylundecane	C13H28	17312-76-4	–	80	34.0	Not tested	2.53E+07	1.1
2-Hydroxy-3,5,6-trimethyl-1,4-benzoquinone	C9H10O3	2913-43-1	–	96	34.6	Not tested	2.19E+07	1.0
Benzamide, N,N-dimethyl-	C9H11NO	611-74-5	+	96	35.2	Yes	4.93E+07	2.2
Farnesane	C15H32	3891-98-3	++	94	35.9	Yes	6.22E+07	2.8
4,4-Dimethyl-2-[(3-nitrophenyl)(1-piperidinyl)methyl]-1(4H)-naphthalenone	C13H26O	1604-34-8	+	96	36.0	Not tested	8.40E+07	3.7
6,10,14-Trimethyl-2-pentadecanol	C18H38O	69729-17-5	–	93	36.2	Not tested	3.63E+07	1.6
Tridecyl vinyl ester carbonic acid	C16H30O3	1000382-54-7	–	93	36.9	Not tested	1.31E+07	0.6
2,6,10-Trimethyltridecane	C16H34	3891-99-4	–	97	37.0	Not tested	1.06E+08	4.7
Pentadecane	C15H32	629-62-9	++	96	37.4	Not tested	1.84E+07	0.8
5,6,7,8-Tetrahydro-2-methyl-1,4-naphthoquinone	C11H12O2	58-26-4	–	88	38.3	Not tested	2.77E+07	1.2
Cis-13-Octadecenal	C18H34O	58594-45-9	+	80	38.7	Not tested	1.11E+07	0.5

Common Name	Formula	CAS#	Hazard*	Match Factor	RT	RT Match	Area	%total area
Hexahydrofarnesol	$C_{15}H_{32}O$	6750-34-1	–	92	39.0	Not tested	4.20E+07	1.9
1,2,3-Trimethylcyclohexane	C_9H_{18}	1678-97-3	–	88	39.2	Not tested	3.01E+07	1.3
Octadec-1-ene	$C_{18}H_{36}$	112-88-9	++	92	39.3	Not tested	1.15E+07	0.5
3,4-Dimethoxybenzenepropanol	$C_{11}H_{16}O_3$	3929-47-3	–	86	39.7	Not tested	2.62E+07	1.2
Pristane	$C_{19}H_{40}$	1921-70-6	+	96	39.8	Yes	1.05E+08	4.7
Phytol	$C_{20}H_{40}O$	150-86-7	+	84	39.9	No	8.86E+07	3.9
Hexyldecanol	$C_{16}H_{34}O$	2425-77-6	–	86	40.0	Not tested	3.43E+07	1.5
1-Tetradecene	$C_{14}H_{28}$	1120-36-1	++	91	40.1	Not tested	2.25E+08	10.0
Hexahydrofarnesol	$C_{15}H_{32}O$	6750-34-1	–	90	40.2	Not tested	4.80E+07	2.1
1,2-Epoxyhexadecane	$C_{16}H_{32}O$	7320-37-8	+	90	40.5	Not tested	2.20E+08	9.8
2,3,6-Trimethylnaphthoquinone	$C_{13}H_{12}O_2$	20490-42-0	+	93	40.6	Not tested	3.42E+07	1.5
Phytane	$C_{20}H_{42}$	638-36-8	–	95	41.2	Not tested	5.77E+07	2.6
Hexahydrofarnesyl acetone	$C_{18}H_{36}O$	502-69-2	–	98	41.5	Yes	2.37E+08	10.5
3,7,11,15-Tetramethyl-1-hexadecyn-3-ol	$C_{20}H_{38}O$	29171-23-1	–	81	42.1	Not tested	2.16E+07	1.0
Phytol	$C_{20}H_{40}O$	150-86-7	+	85	42.6	No	5.87E+07	2.6
3-Methyl-2-(3,7,11-trimethyldodecyl)furan	$C_{20}H_{36}O$	166773-55-3	–	88	42.8	Not tested	1.12E+07	0.5
4-Heptanamine	$C_7H_{17}N$	16751-59-0	+	86	42.9	Not tested	3.08E+07	1.4
5-Methyl-5-(4,8,12-trimethyltridecyl)dihydro-2(3H)-furanone	$C_{21}H_{40}O_2$	96168-15-9	–	91	43.2	Not tested	3.89E+07	1.7
Phytol	$C_{20}H_{40}O$	150-86-7	+	87	43.9	No	1.41E+07	0.6
Hexyldecanol	$C_{16}H_{34}O$	2425-77-6	–	83	44.2	Not tested	3.74E+07	1.7
(+)-Isomenthol	$C_{10}H_{20}O$	23283-97-8	+	79	44.2	Not tested	2.48E+07	1.1

*Hazard assigned to groups using PubChem and Globally Harmonized System (GHS) classification hazard class with the highest hazard class being used for designation (i.e., higher hazard group can include lower hazard class):

"–" = Physical hazard only (H220, H225, H226), or environmental hazard only (H400, H410, H411, H412, H413), or no hazards noted in PubChem;

"+" = Oral acute toxicity (H301, H302), skin corrosion/irritation (H314, H315, H316), skin sensitization (H317), serious eye damage/eye irritation (H319), respiratory tract irritation or narcotic effects from a single exposure with specific target organ toxicity (H335, H336), germ cell mutagenicity (H340, H341), carcinogenicity (H350, H351), reproductive toxicity (H360D, H360FD, H361, H361d, H361f), and/or repeated exposure with specific target organ toxicity (H372, H373); and

“++” = Aspiration hazard (H304) and/or acute inhalation toxicity (H330, H331, H332, or H333).

Notes: Common name taken from Chemspider (http://www.chemspider.com/). Area is the component area after mass spectral deconvolution. %Area is the percentage of reported component area (i.e., sum of %Area equals 100%). Match factor is the automated NIST mass spectral quality factor ranging from 0 to 100 with higher numbers indicating a better match with standard spectra. RT is the retention time of the deconvoluted peak. Some chemicals are repeated at different retention times indicating incompatibility of the chemical with the column leading to multiple peaks, geometric isomers, or inappropriate mass spectral identification.

Supplementary Table S5. Vitamin E oil, second trial: Hazards associated with compounds identified in heated emissions at 250°C

**Common Name**	**Formula**	**CAS#**	**Hazard***	**Match Factor**	**RT**	**RT Match**	**Area**	**%total area**
Trimethylamine	C3H9N	75-50-3	++	99	3.9	Not tested	7.46E+07	1.0
Acetic acid	C2H4O2	64-19-7	+	98	8.1	Yes	1.02E+08	1.4
N-Methyl-1,2-ethanediamine	C3H10N2	109-81-9	+	82	14.6	Not tested	7.49E+07	1.0
Toluene	C7H8	108-88-3	++	98	15.4	Not tested	5.38E+07	0.7
2-Methylheptane	C8H18	592-27-8	++	93	15.9	Yes	5.42E+07	0.7
Benzaldehyde	C7H6O	100-52-7	+	97	22.6	Not tested	8.58E+07	1.2
2-Isooctanone	C8H16O	928-68-7	+	95	22.6	Not tested	1.42E+08	1.9
2,3-Dimethylmaleic anhydride	C6H6O3	766-39-2	+	98	24.6	Not tested	8.39E+07	1.1
4-Methyldecane	C11H24	2847-72-5	–	96	25.9	Not tested	1.34E+08	1.8
Decamethylcyclopentasiloxane	C10H30O5Si5	541-02-6	+	92	30.3	Not tested	5.85E+07	0.8
2,5-Dimethylundecane	C13H28	17301-22-3	–	95	32.0	Not tested	1.19E+08	1.6
2-Tridecyn-1-yl butyrate	C17H30O2	1000299-12-6	–	83	33.3	Not tested	9.01E+07	1.2
6,6-Dimethylundecane	C13H28	17312-76-4	–	81	33.7	Not tested	7.49E+07	1.0
2-Hydroxy-3,5,6-trimethyl-1,4-benzoquinone	C9H10O3	2913-43-1	–	97	34.3	Not tested	1.17E+08	1.6
Dodecamethylcyclohexasiloxane	C12H36O6Si6	540-97-6	+	93	34.9	Not tested	5.75E+07	0.8
Para-dimethylaminobenzaldehyde	C9H11NO	100-10-7	+	76	35.0	Not tested	9.84E+07	1.3
Phytane	C20H42	638-36-8	–	94	35.7	Not tested	1.54E+08	2.1
4,4-Dimethyl-2-[(3-nitrophenyl)(1-piperidinyl)methyl]-1(4H)-naphthalenone	C13H26O	1604-34-8	+	95	35.8	Not tested	2.47E+08	3.4
6,10,14-Trimethyl-2-pentadecanol	C18H38O	69729-17-5	–	93	36.0	Not tested	1.07E+08	1.5
2,6,10-Trimethyltridecane	C16H34	3891-99-4	–	93	36.8	Not tested	2.61E+08	3.6
Pentadecane	C15H32	629-62-9	++	96	37.2	Not tested	6.85E+07	0.9
5-Methoxy-6,7-dimethylbenzofuran	C11H12O2	35355-35-2	–	80	38.1	Not tested	4.90E+07	0.7
Hexahydrofarnesol	C15H32O	6750-34-1	–	94	38.7	Not tested	6.57E+07	0.9
Di(p-tolyl)methane	C15H16	4957-14-6	++	87	38.8	Not tested	7.25E+07	1.0
Hexahydrofarnesol	C15H32O	6750-34-1	–	92	38.9	Not tested	1.63E+08	2.2
Octadec-1-ene	C18H36	112-88-9	++	91	39.1	Not tested	7.44E+07	1.0
6-Hydroxy-4-methoxy-2,3-dimethylbenzaldehyde	C10H12O3	34883-12-0	–	78	39.4	Not tested	1.32E+08	1.8

Common Name	Formula	CAS#	Hazard*	Match Factor	RT	RT Match	Area	%total area
3,4-Dimethoxybenzenepropanol	C11H16O3	3929-47-3	–	80	39.4	Not tested	1.92E+08	2.6
Pristane	C19H40	1921-70-6	+	94	39.6	Yes	3.13E+08	4.3
Phytol	C20H40O	150-86-7	+	84	39.6	Not tested	2.79E+08	3.8
2,10-Dimethyl-6-methyleneundecane	C14H28	33717-93-0	–	89	39.7	Not tested	1.01E+08	1.4
Nonyl tetradecyl ether	C23H48O	1000406-37-6	–	89	39.7	Not tested	1.01E+08	1.4
(2E,7R,11R)-3,7,11,15-Tetramethyl-2-hexadecene	C20H40	14237-73-1	–	89	40.0	Not tested	2.16E+08	3.0
(4Z)-4-Nonadecen-1-yl acetate	C21H40O2	1000131-08-3	–	77	40.2	Not tested	5.54E+08	7.6
2,3,6-Trimethylnaphthoquinone	C13H12O2	20490-42-0	+	93	40.3	Not tested	1.12E+08	1.5
Phytane	C20H42	638-36-8	–	94	40.9	Not tested	2.10E+08	2.9
4-(2,2,6-Trimethylcyclohexyl)-2-butanol	C13H26O	4361-23-3	–	77	41.1	Not tested	3.75E+08	5.1
Hexahydrofarnesyl acetone	C18H36O	502-69-2	–	97	41.1	Yes	5.05E+08	6.9
4-(2,2,6-Trimethylcyclohexyl)-2-butanol	C13H26O	4361-23-3	–	77	41.1	Not tested	3.75E+08	5.1
Phytol	C20H40O	150-86-7	+	85	42.3	Yes	1.57E+08	2.2
Palmitic acid	C16H32O2	57-10-3	+	91	42.7	Not tested	9.62E+07	1.3
1-Pentadecene	C15H30	13360-61-7	++	81	43.8	Not tested	8.99E+07	1.2
Octadecanoic acid	C18H36O2	57-11-4	+	95	47.0	Not tested	1.26E+08	1.7
Ethyl n-octadecanoate	C20H40O2	111-61-5	+	92	47.8	Not tested	7.82E+07	1.1
N-Ethyl-myristamide	C16H33NO	1000408-01-8	–	86	48.6	Not tested	1.14E+08	1.6
5-Methyl-5-(4,8,12-trimethyltridecyl)dihydro-2(3H)-furanone	C21H40O2	96168-15-9	–	93	50.0	Not tested	3.66E+08	5.0
N-Ethyl-myristamide	C16H33NO	1000408-01-8	–	80	51.3	Not tested	1.21E+08	1.7

*Hazard assigned to groups using PubChem and Globally Harmonized System (GHS) classification hazard class with the highest hazard class being used for designation (i.e., higher hazard group can include lower hazard class):

"–" = Physical hazard only (H220, H225, H226), or environmental hazard only (H400, H410, H411, H412, H413), or no hazards noted in PubChem;

"+" = Oral acute toxicity (H301, H302), skin corrosion/irritation (H314, H315, H316), skin sensitization (H317), serious eye damage/eye irritation (H319), respiratory tract irritation or narcotic effects from a single exposure with specific target organ toxicity (H335, H336), germ cell mutagenicity (H340, H341), carcinogenicity (H350, H351), reproductive toxicity (H360D, H360FD, H361, H361d, H361f), and/or repeated exposure with specific target organ toxicity (H372, H373); and

"++" = Aspiration hazard (H304) and/or acute inhalation toxicity (H330, H331, H332, or H333).

Notes: Common name taken from Chemspider (http://www.chemspider.com/). Area is the component area after mass spectral deconvolution. %Area is the percentage of reported component area (i.e., sum of %Area equals 100%). Match factor is the automated NIST mass spectral quality factor ranging from 0 to 100 with higher numbers indicating a better match with standard spectra. RT is the retention time of the deconvoluted peak. Some chemicals are repeated at different retention times indicating incompatibility of the chemical with the column leading to multiple peaks, geometric isomers, or inappropriate mass spectral identification.

Supplementary Table S6. Coconut oil: Hazards associated with compounds identified in heated emissions at 250°C

Common Name	Formula	CAS#	Hazard*	Match Factor	RT	RT Match	Area	%total area
Ethanoic anhydride	C4H6O3	108-24-7	++	91	3.4	No	8.71E+07	1.5
Fluoroethyne	C2HF	2713-09-9	–	76	3.4	Not tested	5.34E+07	0.9
N-Butane	C4H10	106-97-8	+	98	3.7	Not tested	6.82E+07	1.2
2-Aminoisobutyric Acid	C4H9NO2	62-57-7	+	76	4.7	Not tested	9.62E+07	1.7
Pentane	C5H12	109-66-0	++	99	5.3	Not tested	1.07E+08	1.8
Formic acid	CH2O2	64-18-6	+	99	5.4	Yes	8.94E+07	1.5
Butyraldehyde	C4H8O	123-72-8	–	99	7.5	Not tested	1.39E+08	2.4
2-Propanamine, N,2-dimethyl-	C5H13N	14610-37-8	++	82	7.7	Not tested	6.33E+07	1.1
Acetic acid	C2H4O2	64-19-7	+	97	8.4	Yes	2.00E+08	3.4
2-Ethyloxetane	C5H10O	1000386-40-2	–	99	8.6	Not tested	1.09E+08	1.9
Pentan-2-one	C5H10O	107-87-9	+	97	11.5	Not tested	9.75E+07	1.7
n-Pentanal	C5H10O	110-62-3	++	98	11.8	Not tested	1.46E+08	2.5
Propionic acid	C3H6O2	79-09-4	+	88	12.5	Not tested	1.89E+08	3.2
Heptane	C7H16	142-82-5	++	98	13.0	Not tested	1.84E+08	3.2
2-Hexanone	C6H12O	591-78-6	+	97	15.9	Not tested	1.56E+08	2.7
Butyric acid	C4H8O2	107-92-6	+	94	16.2	Not tested	1.65E+08	2.8
Hexanal	C6H12O	66-25-1	+	99	16.4	Not tested	1.68E+08	2.9
Octane	C8H18	111-65-9	++	97	17.3	Not tested	1.33E+08	2.3
Valeric acid	C5H10O2	109-52-4	+	96	19.9	Not tested	1.32E+08	2.3
(1Z,3Z)-1,4-Dimethoxy-1,3-butadiene	C6H10O2	83650-30-0	–	76	20.1	Not tested	8.33E+07	1.4
2-Heptanone	C7H14O	110-43-0	++	94	20.1	Not tested	1.68E+08	2.9
Heptanal	C7H14O	111-71-7	+	97	20.5	Not tested	1.94E+08	3.3
1-Nonane	C9H20	111-84-2	++	98	21.4	Not tested	1.06E+08	1.8
(2E)-2-Heptenal	C7H12O	18829-55-5	+	95	22.5	Not tested	4.95E+07	0.9
Heptan-1-ol	C7H16O	111-70-6	+	88	23.3	Not tested	4.76E+07	0.8
1-Hexanoic acid	C6H12O2	142-62-1	+	90	23.4	Not tested	1.22E+08	2.1
Valeric acid	C5H10O2	109-52-4	+	88	23.4	Not tested	1.07E+08	1.8
2-Octanone	C8H16O	111-13-7	–	95	23.9	Not tested	1.15E+08	2.0

Common Name	Formula	CAS#	Hazard*	Match Factor	RT	RT Match	Area	%total area
Octanal	C8H16O	124-13-0	+	98	24.4	Not tested	1.30E+08	2.2
2,5-Heptanedione	C7H12O2	1703-51-1	–	83	24.4	Not tested	4.34E+07	0.7
Decane	C10H22	124-18-5	++	96	25.0	Not tested	6.57E+07	1.1
N-Heptanoic acid	C7H14O2	111-14-8	+	91	26.7	Not tested	9.22E+07	1.6
1-[Isopropyl(2-methyl-2-propanyl)phosphino]-N,N-dimethylmethanamine	C10H24NP	83718-54-1	–	79	27.4	Not tested	6.85E+07	1.2
2-Nonanone	C9H18O	821-55-6	+	98	27.4	Yes	1.46E+08	2.5
Nonanal	C9H18O	124-19-6	+	99	27.9	Yes	1.56E+08	2.7
Undecane	C11H24	1120-21-4	++	98	28.4	Not tested	6.77E+07	1.2
Caprylic acid methyl ester	C9H18O2	111-11-5	+	95	28.6	Not tested	5.41E+07	0.9
γ-Heptalactone	C7H12O2	105-21-5	–	95	28.7	Not tested	4.34E+07	0.7
2-Methyl-3-pentanyl acetate	C8H16O2	35897-16-6	–	77	29.9	Not tested	4.35E+08	7.5
2-Decanone	C10H20O	693-54-9	–	97	30.7	Not tested	5.09E+07	0.9
(2E)-2-Octenoic acid	C8H14O2	1470-50-4	+	76	32.1	Not tested	7.52E+07	1.3
(2E)-2-Decenal	C10H18O	3913-81-3	+	93	32.7	Not tested	8.43E+07	1.4
δ-Octalactone	C8H14O2	698-76-0	–	98	32.9	Not tested	8.50E+07	1.5
2-Hendecanone	C11H22O	112-12-9	–	96	33.7	Not tested	6.61E+07	1.1
Caprylic anhydride	C16H30O3	623-66-5	+	80	34.5	Not tested	1.05E+08	1.8
Decanoic acid	C10H20O2	334-48-5	+	97	35.0	Not tested	3.06E+08	5.3
Undecenal	C11H20O	2463-77-6	+	96	35.1	Not tested	6.58E+07	1.1
Caprylic anhydride	C16H30O3	623-66-5	+	85	36.1	Not tested	4.99E+07	0.9
γ-Decalactone	C10H18O2	706-14-9	–	92	36.5	Not tested	9.22E+07	1.6
(±)-5-Decanolide	C10H18O2	705-86-2	–	97	36.8	Not tested	4.47E+07	0.8
Capric anhydride	C20H38O3	2082-76-0	+	80	37.4	Not tested	4.65E+07	0.8
Lauric acid	C12H24O2	143-07-7	+	97	37.6	Not tested	6.31E+07	1.1

*Hazard assigned to groups using PubChem and Globally Harmonized System (GHS) classification hazard class with the highest hazard class being used for designation (i.e., higher hazard group can include lower hazard class):

"–" = Physical hazard only (H220, H225, H226), or environmental hazard only (H400, H410, H411, H412, H413), or no hazards noted in PubChem;

"+" = Oral acute toxicity (H301, H302), skin corrosion/irritation (H314, H315, H316), skin sensitization (H317), serious eye damage/eye irritation (H319), respiratory tract irritation or narcotic effects from a single exposure with specific target organ toxicity (H335, H336), germ cell mutagenicity (H340,

H341), carcinogenicity (H350, H351), reproductive toxicity (H360D, H360FD, H361, H361d, H361f), and/or repeated exposure with specific target organ toxicity (H372, H373); and

“++” = Aspiration hazard (H304) and/or acute inhalation toxicity (H330, H331, H332, or H333).

Notes: Common name taken from Chemspider (http://www.chemspider.com/). Area is the component area after mass spectral deconvolution. %Area is the percentage of reported component area (i.e., sum of %Area equals 100%). Match factor is the automated NIST mass spectral quality factor ranging from 0 to 100 with higher numbers indicating a better match with standard spectra. RT is the retention time of the deconvoluted peak. Some chemicals are repeated at different retention times indicating incompatibility of the chemical with the column leading to multiple peaks, geometric isomers, or inappropriate mass spectral identification.

Supplementary Table S7. Medium chain triglyceride (MCT) oil: Hazards associated with compounds identified in heated emissions at 250°C

**Common Name**	**Formula**	**CAS#**	**Hazard***	**Match Factor**	**RT**	**RT Match**	**Area**	**%total area**
2-Nitropropane	C3H7NO2	79-46-9	++	81	3.1	Not tested	5.89E+06	0.8
L-(+)-Alanine	C3H7NO2	56-41-7	–	80	3.4	Not tested	1.62E+07	2.3
N-Butane	C4H10	106-97-8	+	97	3.7	Not tested	6.19E+06	0.9
Ethanol	C2H6O	64-17-5	–	99	4.2	Yes	4.51E+06	0.6
Ethanoic anhydride	C4H6O3	108-24-7	++	93	4.7	No	1.74E+07	2.5
Propionaldehyde	C3H6O	123-38-6	+	90	4.7	Not tested	1.84E+07	2.6
Pentane	C5H12	109-66-0	++	98	5.3	Not tested	8.97E+06	1.3
Formic acid	CH2O2	64-18-6	+	98	5.5	Yes	1.09E+07	1.6
Butyraldehyde	C4H8O	123-72-8	–	99	7.5	Not tested	2.07E+07	2.9
Butanone	C4H8O	78-93-3	+	98	7.7	Not tested	1.90E+07	2.7
Acetic acid	C2H4O2	64-19-7	+	98	7.9	Yes	3.09E+07	4.4
2-Ethyloxetane	C5H10O	1000386-40-2	–	99	8.6	Not tested	1.16E+07	1.6
Pentan-2-one	C5H10O	107-87-9	+	98	11.5	Not tested	1.86E+07	2.6
n-Pentanal	C5H10O	110-62-3	++	99	11.8	Not tested	2.00E+07	2.8
Propionic acid	C3H6O2	79-09-4	+	87	12.0	Not tested	1.15E+07	1.6
Heptane	C7H16	142-82-5	++	98	13.0	Not tested	1.61E+07	2.3
Butyric acid	C4H8O2	107-92-6	+	95	15.8	Not tested	1.73E+07	2.5
1,1-Dimethyl-2-propanol	C5H12O	598-75-4	++	65	15.8	Not tested	5.69E+06	0.8
2-Hexanone	C6H12O	591-78-6	+	99	16.0	Not tested	2.41E+07	3.4
Hexanal	C6H12O	66-25-1	+	98	16.4	Not tested	2.06E+07	2.9
Octane	C8H18	111-65-9	++	98	17.3	Not tested	9.06E+06	1.3
Valeric acid	C5H10O2	109-52-4	+	81	19.6	Not tested	1.28E+07	1.8
γ-Hydroxybutyric acid	C4H8O3	591-81-1	+	86	19.7	Not tested	6.61E+06	0.9
2-Heptanone	C7H14O	110-43-0	++	96	20.1	Not tested	3.26E+07	4.6
Heptanal	C7H14O	111-71-7	+	98	20.5	Not tested	2.52E+07	3.6
1-Nonane	C9H20	111-84-2	++	99	21.4	Not tested	1.02E+07	1.4
γ-Valerolactone	C5H8O2	108-29-2	–	97	21.5	Not tested	5.78E+06	0.8
1-Hexanoic acid	C6H12O2	142-62-1	+	94	23.2	Not tested	7.90E+06	1.1

Common Name	Formula	CAS#	Hazard*	Match Factor	RT	RT Match	Area	%total area
2-Octanone	C8H16O	111-13-7	–	96	23.9	Not tested	1.33E+07	1.9
Octanal	C8H16O	124-13-0	+	98	24.4	Not tested	1.05E+07	1.5
2,5-Heptanedione	C7H12O2	1703-51-1	–	86	24.4	Not tested	5.64E+06	0.8
Heptane	C7H16	142-82-5	++	89	25.0	Not tested	6.89E+06	1.0
γ-Caprolactone	C6H10O2	695-06-7	–	96	25.3	Not tested	6.82E+06	1.0
N-Heptanoic acid	C7H14O2	111-14-8	+	96	26.5	Not tested	8.24E+06	1.2
2-Nonanone	C9H18O	821-55-6	+	98	27.4	Yes	2.12E+07	3.0
Nonanal	C9H18O	124-19-6	+	98	27.9	Yes	1.22E+07	1.7
Caprylic acid methyl ester	C9H18O2	111-11-5	+	90	28.6	Not tested	7.09E+06	1.0
γ-Heptalactone	C7H12O2	105-21-5	–	93	28.7	Not tested	8.15E+06	1.2
Caprylic acid	C8H16O2	124-07-2	+	98	29.7	Not tested	6.66E+07	9.5
2(3H)-Furanone, 5-butyldihydro-	C8H14O2	104-50-7	–	93	32.1	Not tested	3.41E+07	4.8
δ-Octalactone	C8H14O2	698-76-0	–	97	32.9	Not tested	1.96E+07	2.8
Capric acid methyl ester	C11H22O2	110-42-9	–	94	34.4	Not tested	4.54E+06	0.6
Caprylic anhydride	C16H30O3	623-66-5	+	80	34.5	Not tested	1.11E+07	1.6
Decanoic acid	C10H20O2	334-48-5	+	97	35.0	Not tested	1.64E+07	2.3
Caprylic anhydride	C16H30O3	623-66-5	+	85	36.1	Not tested	4.93E+06	0.7
γ-Decalactone	C10H18O2	706-14-9	–	94	36.5	Not tested	2.10E+07	3.0
(±)-5-Decanolide	C10H18O2	705-86-2	–	95	36.8	Not tested	1.07E+07	1.5

*Hazard assigned to groups using PubChem and Globally Harmonized System (GHS) classification hazard class with the highest hazard class being used for designation (i.e., higher hazard group can include lower hazard class):

"–" = Physical hazard only (H220, H225, H226), or environmental hazard only (H400, H410, H411, H412, H413), or no hazards noted in PubChem;

"+" = Oral acute toxicity (H301, H302), skin corrosion/irritation (H314, H315, H316), skin sensitization (H317), serious eye damage/eye irritation (H319), respiratory tract irritation or narcotic effects from a single exposure with specific target organ toxicity (H335, H336), germ cell mutagenicity (H340, H341), carcinogenicity (H350, H351), reproductive toxicity (H360D, H360FD, H361, H361d, H361f), and/or repeated exposure with specific target organ toxicity (H372, H373); and

"++" = Aspiration hazard (H304) and/or acute inhalation toxicity (H330, H331, H332, or H333).

Notes: Common name taken from Chemspider (http://www.chemspider.com/). Area is the component area after mass spectral deconvolution. %Area is the percentage of reported component area (i.e., sum of %Area equals 100%). Match factor is the automated NIST mass spectral quality factor ranging from 0

to 100 with higher numbers indicating a better match with standard spectra. RT is the retention time of the deconvoluted peak. Some chemicals are repeated at different retention times indicating incompatibility of the chemical with the column leading to multiple peaks, geometric isomers, or inappropriate mass spectral identification.
